# Altered Regulation of Striatal Neuronal *N*-Methyl-D-Aspartate Receptor Trafficking by Palmitoylation in Huntington Disease Mouse Model

**DOI:** 10.3389/fnsyn.2019.00003

**Published:** 2019-02-21

**Authors:** Rujun Kang, Liang Wang, Shaun S. Sanders, Kurt Zuo, Michael R. Hayden, Lynn A. Raymond

**Affiliations:** ^1^Department of Psychiatry, Brain Research Centre and Djavad Mowafaghian Centre for Brain Health, The University of British Columbia, Vancouver, BC, Canada; ^2^Department of Medical Genetics, Centre for Molecular Medicine and Therapeutics, Child and Family Research Institute, The University of British Columbia, Vancouver, BC, Canada

**Keywords:** Huntington disease (HD), NMDAR palmitoylation, palmitoyl acyltransferase (PAT), huntingtin interacting protein 14 (HIP14), huntingtin interacting protein 14-like (HIP14L)

## Abstract

*N*-methyl-D-aspartate receptors (NMDARs) play a critical role in synaptic signaling, and alterations in the synaptic/extrasynaptic NMDAR balance affect neuronal survival. Studies have shown enhanced extrasynaptic GluN2B-type NMDAR (2B-NMDAR) activity in striatal neurons in the YAC128 mouse model of Huntington disease (HD), resulting in increased cell death pathway activation contributing to striatal vulnerability to degeneration. However, the mechanism(s) of altered GluN2B trafficking remains unclear. Previous work shows that GluN2B palmitoylation on two C-terminal cysteine clusters regulates 2B-NMDAR trafficking to the surface membrane and synapses in cortical neurons. Notably, two palmitoyl acyltransferases (PATs), zDHHC17 and zDHHC13, also called huntingtin-interacting protein 14 (HIP14) and HIP14-like (HIP14L), directly interact with the huntingtin protein (Htt), and mutant Htt disrupts this interaction. Here, we investigated whether GluN2B palmitoylation is involved in enhanced extrasynaptic surface expression of 2B-NMDARs in YAC128 striatal neurons and whether this process is regulated by HIP14 or HIP14L. We found reduced GluN2B palmitoylation in YAC128 striatum, specifically on cysteine cluster II. Consistent with that finding, the palmitoylation-deficient GluN2B Cysteine cluster II mutant exhibited enhanced, extrasynaptic surface expression in striatal neurons from wild-type mice, mimicking increased extrasynaptic 2B-NMDAR observed in YAC128 cultures. We also found that HIP14L palmitoylated GluN2B cysteine cluster II. Moreover, GluN2B palmitoylation levels were reduced in striatal tissue from HIP14L-deficient mice, and siRNA-mediated HIP14L knockdown in cultured neurons enhanced striatal neuronal GluN2B surface expression and susceptibility to NMDA toxicity. Thus, altered regulation of GluN2B palmitoylation levels by the huntingtin-associated PAT HIP14L may contribute to the cell death-signaling pathways underlying HD.

## Introduction

*N*-methyl-D-aspartate (NMDA) receptors are a subclass of ionotropic glutamate receptors that play critical roles in excitatory synaptic transmission. Abnormal NMDA receptor (NMDAR) function is implicated in many neuropsychiatric disorders and neurodegenerative diseases (Lipton and Rosenberg, 1994; Aarts et al., 2002; Arundine and Tymianski, 2003; Hardingham and Bading, 2010; Malinow, 2013). The activation of NMDA receptors in different plasma membrane microdomains leads to distinct cellular signaling pathways: synaptic NMDAR activity provides neuroprotection (Hardingham et al., 2002; Ivanov et al., 2006; Leveille et al., 2008), whereas extrasynaptic NMDAR activity is linked to cell death pathways (Aarts et al., 2002; Hardingham et al., 2002; Arundine and Tymianski, 2003). In addition to extrasynaptic localization, subunit composition has been proposed to play a role; GluN2B-containing NMDARs have been shown to facilitate neuronal death more than GluN2A-NMDARs (Liu et al., 2007; Martel et al., 2012).

Several lines of evidence suggest a role for altered NMDAR function in the pathogenesis of the autosomal dominant-inherited neurodegenerative disorder, HD (Levine et al., 1999; Li, 2004; Zeron et al., 2004; Graham et al., 2009). HD is caused by a CAG repeat expansion >35 in the gene for huntingtin (Htt) (Lin et al., 1993), which results in prominent degeneration of striatal medium-sized spiny neurons (MSNs) (Vonsattel and Difiglia, 1998). Progressive striatal atrophy and behavioral manifestations of HD are reproduced in the YAC128 transgenic mouse model, expressing the full-length human genomic DNA for mutant Htt (mHtt) (Slow et al., 2003). Previous studies in YAC128 and other HD mouse models demonstrate increased striatal neuronal NMDAR current and excitotoxicity (Levine et al., 1999; Li, 2004; Zeron et al., 2004), associated with altered NMDAR surface membrane trafficking (Fan et al., 2007), prior to onset of motor deficits (Milnerwood and Raymond, 2007; Graham et al., 2009). Increased GluN2B-type NMDAR (2B-NMDAR) surface expression was found localized to extrasynaptic sites in YAC128 mice striatal neurons within one month of birth (Milnerwood et al., 2010), resulting in a shift toward reduced cell survival and enhanced cell death signaling in the striatum (Okamoto et al., 2009; Milnerwood et al., 2010, 2012; Dau et al., 2014), including reduced nuclear phospho-cyclicAMP Response Binding protein (pCREB) and elevated expression of activated p38 MAP Kinase (Cowan et al., 2008; Xu et al., 2009; Milnerwood et al., 2010, 2012; Fan et al., 2012; Gladding et al., 2012, 2014). Moreover, we found that the pro-death signaling in YAC128 striatum can be reversed by treatment of mice from 2 to 4 months of age (Milnerwood et al., 2010; Dau et al., 2014) with low-dose memantine, which selectively blocks activity of extrasynaptic and not synaptic NMDAR (Okamoto et al., 2009; Xia et al., 2010). The results of these studies and previously published work summarized above support the idea that extrasynaptic 2B-NMDAR stimulation preferentially activates cell death signaling pathways (Aarts et al., 2002; Hardingham et al., 2002; Arundine and Tymianski, 2003; Ivanov et al., 2006; Leveille et al., 2008; Hardingham and Bading, 2010; Parsons and Raymond, 2014). Therefore, it is important to understand the regulatory mechanism(s) of altered NMDAR surface membrane trafficking in striatal neurons in YAC HD mice.

Previous studies have shown that the surface membrane trafficking of NMDARs is regulated by palmitoylation (Hayashi et al., 2009; Thomas and Huganir, 2013). Palmitoylation is a reversible posttranslational lipid modification that attaches the 16-carbon palmitic acid to cysteines by thioester linkage, functioning to tether proteins to membranes or sort proteins to particular cellular microdomains (El-Husseini and Bredt, 2002; Fukata and Fukata, 2010). Palmitate attachment to certain synaptic proteins is a dynamic process that regulates protein trafficking and function (Kang et al., 2008; Fukata et al., 2013, 2015). Protein palmitoylation is catalyzed by a family of 23 mammalian DHHC domain-containing PATs (Fukata et al., 2004; Huang et al., 2004) and only two major neuronal PATs, zDHHC17 and zDHHC13, are associated with the Htt (Singaraja et al., 2002). zDHHC17 was first identified as HIP14 (Singaraja et al., 2002) and regulates the palmitoylation and trafficking of several synaptic proteins, including PSD-95, SNAP-25, GAD-65, synaptotagmin I and huntingtin (Htt) (Huang et al., 2004). Wild-type Htt modulates the enzymatic activity and palmitoylation of HIP14 (Huang et al., 2011); as well, HIP14’s enzymatic activity for synaptic substrate SNAP-25 was found to be reduced in whole brain lysates from YAC128 HD mice compared with those from wild-type mice, suggesting that mutant Htt (mHtt) expression reduces HIP14 function (Singaraja et al., 2011). Another major PAT for the Htt protein, zDHHC13 was identified in a database search for HIP14 homologs. zDHHC13, or HIP14-like (HIP14L), which is in the same phylogenetic tree with zDHHC17 (HIP14) (Singaraja et al., 2002; Fukata and Fukata, 2010), also interacts with wild-type Htt but less strongly with mHtt protein (Sutton et al., 2013; Sanders et al., 2014). Genetic ablation of either HIP14 or HIP14L recapitulates many features of HD, including striatal atrophy and motor deficits; the phenotype of these two mouse models is thought to result from underpalmitoylation of their cellular substrates (Singaraja et al., 2011; Sutton et al., 2013; Sanders and Hayden, 2015).

In YAC128 HD mice, the increased 2B-NMDAR steady-state surface expression is localized to extrasynaptic membrane sites and is largely a result of enhanced forward trafficking to the surface (Fan et al., 2007; Milnerwood et al., 2010, 2012). GluN2B can be palmitoylated on two cysteine (Cys or C) clusters in the C-terminal domain (Hayashi et al., 2009). Mutation of cysteines to serines (Ser or S) in the membrane-proximal cluster (Cys cluster I, 3CS mutant: C849S, C854S, C871S) leads to a significant reduction in 2B-NMDAR surface expression and synaptic current in cultured cortical neurons (Hayashi et al., 2009; Mattison et al., 2012). In contrast, mutations in the second cysteine cluster of GluN2B (Cys cluster II, 5CS mutant: C1215S, C1218S, C1239S, C1242S, and C1245S) resulted in an increase in NMDAR surface expression, but not NMDAR-mediated synaptic currents in cortical neurons (Hayashi et al., 2009; Mattison et al., 2012), suggesting that palmitoylation of cluster I regulates incorporation of functional 2B-NMDARs into synapses whereas reduced palmitoylation of cluster II may increase the pool of extrasynaptic receptors in cultured cortical neurons. However, these effects were observed in glutamatergic neurons; whether palmitoylation of GluN2B affects its surface incorporation in GABAergic neurons such as striatal neurons is unknown. In this study, we investigated whether palmitoylation of GluN2B contributes to increased 2B-NMDAR extrasynaptic localization in striatal neurons from YAC128 HD mice and explored a role for Htt-associated PATs (HIP14 and HIP14L) in regulating GluN2B palmitoylation.

## Materials and Methods

### Animals

YAC128, HIP14-/- and HIP14L-/- mouse lines were previously characterized (Slow et al., 2003; Singaraja et al., 2011; Sutton et al., 2013). Animals were housed and maintained according to the Canadian Council on Animal Care at the UBC Faculty of Medicine Animal Resource Unit. All procedures were approved by the University of British Columbia Committee on Animal Care.

### Primary Neuronal Co-culture and Transfection

Neuronal cultures were prepared from either E17/E18 of both sex FVB/N and YAC128 mice. Cortical and striatal neurons were dissociated by enzymatic digestion with trypsin followed by brief mechanical trituration. Cells were plated on poly-D lysine (Sigma) pre-treated 10 cm plates or 24-well plates with glass coverslips (12 mm in diameter), and then were maintained in Neurobasal media (NBM, Invitrogen) supplemented with B27 (Invitrogen), penicillin, streptomycin, and L-glutamine as described before (Brewer et al., 1993; Milnerwood et al., 2012). DNA constructs for GluN2B WT, GluN2B 5CS and GluN2B 3CS, each tagged at the N-terminus with GFP, were kindly provided by Dr. Richard Huganir, Johns Hopkins University. 3–5 μg of one of these endonuclease-free DNAs were electroporated into striatal neurons, which were then plated with untransfected cortical neurons at a 1:1 ratio (1.1 × 10^5^ striatal cells + 1.1 × 10^5^ cortical cells/well of 24-well plates) as described before (Kaufman et al., 2012; Milnerwood et al., 2012). These striatal-cortical (MSN-CTX) co-cultures were used for all experiments unless otherwise indicated.

### Immunocytochemistry

Since GFP fluorescence on GFP-GluN2B expressing cells is very dim after fixation, we amplified the signal by immunostaining with an anti-GFP antibody. Staining for surface versus internal GFP-GluN2B staining was performed as previously described (22, 27, and 56). Briefly, pairs of DIV 17–18 FVB/N and YAC128 cortico-striatal cultures transfected with the different GFP-GluN2B constructs were incubated for 10 min at 37°C with anti-chicken GFP polyclonal antibody (AbCam; 1:1000) in conditioned media for live staining; the cells were fixed in 4% PFA with 4% sucrose for 10 min and then washed 3 times with PBS. Cells were then incubated with anti-chicken secondary antibody conjugated to Alexa 488 fluorophores (1:2000 in PBS) for 1 h at room temperature (RT). This live staining allows anti-GFP antibody to recognize the extracellular epitope on surface GFP-GluN2B without binding to internal GFP GluN2B (Milnerwood et al., 2012). Then, for internal GluN2B staining, cells were subsequently permeabilized by adding 0.3% Triton X-1000 in PBS (PBST), then incubated with anti-chicken GFP antibody (1:1000 in PBST) followed by incubation with secondary antibody conjugated to the Alexa 568 fluorophore (1:2000 in PBST) for 1 h at RT. Since surface GFP-GluN2B is already occupied by anti-GFP antibodies and the green secondary, the red secondary binds only to anti-GFP that is bound to internal GFP-GluN2B. Controls for surface GluN2B (green) and internal GluN2B (red) staining were performed in non-transfected and GFP-GluN2B transfected neurons (with and without permeabilization) described as before (Gladding et al., 2012; Kaufman et al., 2012). No significant dendritic green or red fluorescent signals were observed when the staining protocol was performed on non-transfected cells. Moreover, in GFP-GluN2B transfected cells there was no observable red staining in the absence of permeabilization, indicating that the red staining is specific for internal GFP-GluN2B (data not shown). For labeling of PSD-95 and VGLUT1, following incubation of live cells with anti-GFP antibodies to detect surface GluN2B as described above, cells were subsequently permeabilized with methanol for 5 min at -20°C, rinsed 3 times with PBST, then incubated with primary antibodies for PSD-95 (Thermo Scientific; 1:1000) and VGLUT1 (Millipore; 1:2000) at 4°C overnight, washed 3 times with PBST, and incubated with secondary antibodies conjugated to Alexa 568 fluorophore and AMCA (1:100) for 1 h at RT. Coverslips were then mounted on slides (Frost Plus; Fisher) with Fluoromount-G (Southern Biotechnology) and images were taken using a Zeiss Axiovert 200M fluorescence microscope.

### Microscopy and Image Analysis

For GluN2B surface/internal expression (green/red fluorescence ratio) analysis, images (63×, 12 *z*-stacks of 0.4 μm) were acquired, all exposure times were kept at the same level within experiments and analysis was conducted on unprocessed (raw) images. *Z*-stack images were flattened, using the extended focus projection function (objective was 1.4 NA; Zeiss, ZEN system), from 4 to 5 focal planes containing all of the visible dendritic surface staining. GluN2B surface and internal expression fluorescence were quantified in ImageJ (National Institutes of Health) as mean intensity within three regions of interest (ROI), which included one primary dendrite and two secondary dendrites. The mean intensity was measured, subtracting background beside the individual ROI, and the ratio of surface fluorescence (green) to internal (red) signal was calculated for each cell. The representative images were processed with the default deconvolution program in ZEN2012 system (Zeiss) to improve the resolution. For localization analysis, images (63×, 12 *z*-stacks of 0.4 μm) were acquired, and extended focus projections were created from 4 to 5 focal planes containing all of the visible dendritic surface staining. For cluster intensity, density and localization, images were thresholded at a level of background mean intensity plus 5 standard deviations with the experimenter blind to condition, and the puncta quantification was measured in regions of interest around one primary dendrite and two secondary dendrites. Co-localization was calculated using an ImageJ plug-in^1^. Statistical analysis was done with ANOVA and *t-*tests, which were conducted in Prism 4 software (GraphPad) as detailed in the text. The representative images were processed with the default deconvolution program in ZEN2012 system (Zeiss) to improve the resolution.

### Acyl-Biotin Exchange (ABE) Assay/Western Blot Analysis

Brain tissues from male FVB/N wild-type, YAC128, HIP14-/- and HIP14L-/- mice were flash frozen in liquid nitrogen and preserved at -80°C until they were forwarded to ABE/western analysis. The striatum and cortex obtained from male FVB/N and YAC128 mice brains were homogenized in lysis buffer containing 150 mM NaCl, 50 mM Tris (pH7.4), and 5 mM EGTA, to which 50 mM NEM (Sigma), 0.5 mM PMSF and 1 tablet (Roche) protease inhibitor cocktail (leupeptin, chymostatin, pepstatin, aprotinin) per 10 ml buffer were added; 1.7% Triton X-100 and 0.2% SDS were subsequently added and then incubated with end-over-end rotation at 4°C for 1 h. Particulates and unbroken cells were removed by centrifugation (13,200 rpm, 4°C, 10 min). The protein extracts were subjected to the three-step Chloroform–methanol (CM) precipitation as described before (Wan et al., 2007; Kang et al., 2008). After completely drying the pellet in air, pellets were dissolved in lysis buffer to which the following were added: 0.2% SDS, 0.2% triton X-100 and 50 mM NEM, then subjected to end-over-end rotation at 4°C overnight. The next day, after 2 times CM precipitation, one half of the sample was treated with 1 M hydroxylamine (HAM) (Sigma) in lysis buffer for 1 h to remove palmitate from Cys residues (+HAM) and the other half was the control sample (-HAM). Simultaneously, samples were treated with the biotinylated Cys-crosslinking reagent 1mM HPDP-biotin (Thermo Scientific). After two-times CM precipitation to completely remove HAM and HPDP-biotin, biotinylated proteins were then isolated by pull-down using streptavidin sepharose (GE) beads as described before (Wan et al., 2007; Kang et al., 2008).

**FIGURE 1:**
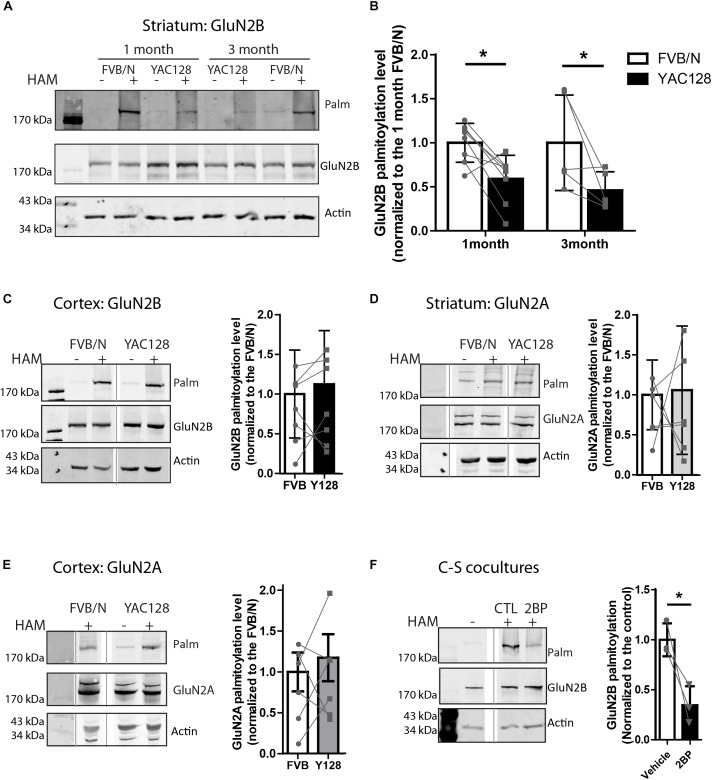
Palmitoylation of GluN2B but not GluB2A is significantly reduced in YAC128 striatum. Brain striatal and cortex tissues were dissected from FVB/N wild-type and YAC128 mice, rapidly frozen by liquid nitrogen, and saved at –80°C before forwarding to ABE/western analysis. Each panel of image data shows results from one representative gel and the dividing line(s) indicate where lanes were removed for ease of comparing genotypes from specific tissues and ages. **(A)** Representative blot shows that the palmitoylation of GluN2B as a proportion to total was significantly reduced in striatal tissues from 1- and 3 month- old YAC128 mice compared to FVB/N (wild-type). The HAM – and + indicate lysates treated without/with hydroxylamine (HAM); lysates without HAM treatment are controls for non-specific protein precipitation by streptavidin-linked beads and only HAM+ lanes were analyzed. Quantification of GluN2B palmitoylation was measured as the band intensity in the purified palmitoyl-proteins (top panel: Palm) ratio to the level of the corresponding unpurified total protein extracts (middle panel: total GluN2B); actin expression reflects protein loading control (lower panel). The graph analysis **(B)** indicates the corresponding GluN2B palmitoylation level for YAC128 normalized to FVB/N wild-type control within the same experiment. Data are presented for eight independent experiments from 1 month-old and five independent experiments from 3 month-old FVB/N and YAC128 mice. Bars represent means ± SD ^∗^*p* < 0.05 (two-way ANOVA, *p* = 0.0409 for genotype; *p* = 0.5235 for interaction and age). Data points from the same blot are connected by lines. **(C)** The palmitoylation of GluN2B was not altered in cortical tissues from 1 month-old YAC128 mice. The graph analysis, generated from raw data as in **(B)**, is from 5 independent experiments. **(D,E)** The GluN2A palmitoylation level was similar in both striatal and cortical tissues from 1 month-old YAC128 and FVB/N control mice. Data are presented from six independent experiments. The protein ladder lane image was contrast-enhanced using Adobe Photoshop in **(E)**. **(F)** Rapid turnover rate of palmitate on GluN2B. MSN-CTX co-cultures at DIV 14 were treated with 100 μM 2-bromo-palmitate (2BP) for 5 h and then harvested for ABE/western analysis. Representative blots show significant reduction of palmitoylation of GluN2B in the absence of any protein expression change. Graph was generated with data from three independent experiments. Bars represent means ± SD ^∗^*p* < 0.05 (paired *t*-test).

For western blot analysis, the protein samples were resuspended in Laemmli buffer (4% SDS, 20% glycerol, 0.004% bromphenol blue, 0.125 M Tris HCl, pH 6.8) with 10 mM DTT, and samples were analyzed by SDS-PAGE (NuPAGE 4–12% Bis-Tris Gel, Invitrogen). SDS-PAGE gels were transferred onto nitrocellulose membranes (Amersham, 0.5A for 90 min at 4°C) and probed with specific antibodies: anti-GluN2B (Millipore, anti-mouse MA1-2014, 1:1000), anti-GluN2A (Thermo Scientific, 1:1000), anti-GFP (AbCam, 1:1000), anti-Tubulin (Sigma, 1:5000) and anti-Actin (Sigma, 1:2000). Primary antibodies were applied overnight in Odyssey blocking buffer (Li-COR Bioscience). The secondary antibodies (Rockland, anti-rabbit IRD800; Invitrogen, anti-Mouse Alexa Fluor 680) were used at a dilution of 1:10,000. The immunoblots were scanned using the Odyssey Infrared Imaging System (Li-COR Biosciences), and band intensity was quantified by Image J software (NIH). Palmitoylation levels, measured from the band intensity of purified palmitoyl-protein samples (palm), were normalized to the band intensity measured from the corresponding unpurified extracts (Total). Actin expression, corresponding to the unpurified extracts (Total), is used as an internal standard for any variability in protein expression. Palmitoylation level in the control condition in the HAM+ lanes was averaged for normalization, and quantitative changes in palmitoylation level for other conditions calculated as a ratio to the control. Although the absolute value of the band intensities and ratio to the control condition varied between experiments based on the batch of antibodies and exposure times used, we compared between genotypes within each experiment, in which protein was loaded from WT and YAC128 mouse brain tissues onto the same gel and taken through the western blot protocol on the same blot. Thus, despite large standard deviations in the mean values for our data sets, significant differences were revealed by over-plotting the individual ratio points connected by lines to indicate the genotype pairs (e.g., Figure 1B). Significant differences were determined using Prism software (Graphpad, Inc.). Significant differences between genotype and ages were assessed by the two-way ANOVA test and direct comparisons between genotype were made by paired two-tail Student’s *t*-test (2-tailed, Mann–Whitney *U*). Each of the representative western blot panels shown in the figures is an example from one original blot, and the dividing line(s) in each panel indicate where some lanes on the original blot were removed in order to more clearly display a side-by-side comparison between genotypes of results from particular tissues or ages; a line between the protein ladder and the rest of the blot indicates that the protein ladder lane was subjected to contrast enhancement for improved visualization. In some experiments (i.e., Figure 1D,E, 6A,C) extracts from some of the matched HAM- samples were run on a different gel because of the need to directly compare multiple experimental conditions for HAM+ samples on the same gel; in those figures, those particular HAM- samples are not shown.

### COS-7 Cell Transfection

COS-7 cells were co-transfected with GFP-tagged GluN2B WT or GluN2B 5CS or GluN2B 3CS together with HA-tagged GluN1-1A, combined with either pCINeo empty vector or a HIP14-Flag (Yanai et al., 2006) or HIP14L-Flag construct (Huang et al., 2009), in either 6- or 24-well plates. The co-transfection ratio of DNAs (GluN2B: HA-GluN1-1A: pCINeo/HIP14-Flag or pCINeo/HIP14L-Flag) was 4:4:1. To inhibit proteasome degradation, 100 μM MG-132 (Selleckchem) was added to cells 24 h after transfection. After 36–48 h of overexpression, cells in the 6-well plates were harvested and forwarded to the ABE/western blot assay and co-immunoprecipitation; cells in the 24-well plates were fixed with 4% PFA for 10 min, then incubated with antibodies raised against GFP (Abcam; 1:2000), Flag (Sigma; 1:1000) and GOLPH4 (AbCam; 1:1000), and after washing with PBS-T, cells were incubated with secondary antibody conjugated to Alexa 568 (1:1000) and AMCA (1:100) for 1 h at room temperature (RT). Images were acquired by using a 63× objective affixed to a Zeiss inverted microscope and ZEN2012 system software. Line scan analysis was performed for perinuclear region accumulation of the various GFP-tagged GluN2B constructs with Golgi marker, GOLPH4. Briefly, perinuclear regions were line scanned for each channel using ImageJ, perinuclear region intensity profiles were incorporated into excel 2D-line graphs, and intensity peak registration of the GFP (GluN2B) green channel with GOLPH4 blue channel was assessed. Cells showing co-registration of more than 50% of the peaks in the GFP and GOLPH4 channels were defined as “positive” for GluN2B-GOLPH4 perinuclear region colocalization; only peaks showing elevations in intensity sustained over >10 microns of the line scan were included in the analysis. Using this approach, the percentage of cells showing GluN2B-GOLHP4 perinuclear co-localization was calculated from line scans of 30 randomly selected cells per condition. Statistical analysis was done with two-way ANOVA, which was conducted in Prism 4 software (GraphPad).

### Co-immunoprecipitation

COS-7 cells were lysed in ice-cold buffer (150 mM NaCl, 50 mM Tris pH7.4, 5 mM EGTA, 0.2% SDS, 1% Triton X-100, one protease inhibitor tablet/10 ml, 10 mM PMSF). Cell lysates were rotated at 4°C for 1 h before the insoluble materials were removed by centrifugation at 13,200 rpm for 15 min. Lysates were precleared by incubation with protein A sepharose beads (GE Healthcare) for 45 min at 4°C with rotation. Precleared lysates were then incubated with anti-GFP (5 μg, rabbit, in-house) antibody with rotation, at 4°C overnight. Proteins in precipitates were heated in 2× sample buffer and then applied to SDS-PAGE. After 1 h transfer of protein to nitrocellulose membrane, western blot was probed with anti-Flag antibody (Sigma, 1:1000) and anti-GFP antibody (AbCam, 1:1000).

### Calpain Cleavage, Btn-BMCC Labeling

Striatal tissues were dissected from 2 months old wild type (FVB/N) and YAC128 mice and fresh frozen at -80°C before applying to the assay. After thawing on ice, samples were homogenized and processed for immunoprecipitation as described above. The protein concentration was determined after preclearing. The first immunoprecipitation was processed by incubation of 5 mg of precleared lysates with 20–25 μg anti-GluN2B N-terminal antibody (Alomone: AGC-003), then after 3 times washing, the beads were divided into two equal portions and forwarded to the calpain cleavage assays. In brief, immunoprecipitates from both portions were diluted in 400 μl calpain reaction buffer (20 mM HEPES pH7.6, 10 mM KCl, 1.5 mM MgCl_2_, 1 mM dithiothreitol) with 10 nM of recombinant calpain-1 (EMD-Millipore, 208712) and 2 mM CaCl_2_ for 10 min at RT, except one half was pre-treated with 100 μM of calpain inhibitor CI-III (EMD Millipore, 208722). The calpain cleavage reactions were stopped by adding 100 μM of calpain inhibitor CI-III (EMD Millipore, 208722) and centrifuging immediately for 5 s at 13,200 rpm. Supernatants were transferred to new tubes and forwarded to the second immunoprecipitation step, with 10 μg of a GluN2B C-terminus-targeted antibody (a.a. 1437–1456, EMD Millipore, 06-600, anti-rabbit). After 3 times washing, the beads were washed with Stringent buffer (0.5 M NaCl, 5 mM EDTA, 50 mM Tris pH7.4, 0.1% SDS) with 50 mM NEM and one tablet protease inhibitor and forwarded to the Btn-BMCC palmitoylation assay as described before (Yanai et al., 2006; Huang et al., 2011). Because the 10 min incubation with calpain in the absence of calpain inhibitor resulted in complete loss of C-terminal GluN2B secondary to further proteolysis, only the products of the calpain cleavage reaction that contained the inhibitor could be analyzed. After NEM blockade steps, C-terminal fragment-containing samples were divided into two parts, one third of each sample was reserved as the HAM- lysate and two thirds were treated with HAM (HAM+ lysates); these two sets of samples were then processed in parallel through all subsequent steps. Palmitoylation of the cluster II-containing C-terminal fragment of GluN2B was detected by probing with streptavidin Alexa Fluor^®^680 conjugate (Invitrogen, 1:10,000) and total GluN2B was probed with anti-mouse C-terminal-targeted antibody (Thermo Fisher Scientific, MA1-2014). In parallel, cleaved N-terminal fragments were carried over to ABE assay as described above, which will stop further calpain activity. Palmitoylation of N-terminal fragments was detected by probing with GluN2B N-terminal specific antibody (Alomone, AGC-003).

### Hip14-ASO and HIP14L siRNA Treatment

A phosphorothioate backbone ASO with five 2′*O*-methoxyethyl ribose sugars in the wings was designed against the HIP14 3′UTR by Ionis Pharmaceuticals (515084, 5′-TGCTTTATTTTCAGACCGTG-3′). *Hip14*-ASO was resuspended in sterilized PBS to a concentration of 0.5–1 μM. The potency of this *Hip14*-ASO was tested by bath application into the media of day *in vitro* (DIV) 3 FVB/N mouse primary cortical neurons, which freely take up ASOs from the media (Carroll et al., 2011). Neurons were harvested 7 days post treatment and lysed using a 1% SDS/TEEN buffer (50 mM Tris pH 7.5, 1 mM EDTA, 1 mM EGTA, and 150 mM NaCl) with 1x Roche Complete Protease Inhibitor cocktail and applied on a NuPAGE Novex 4–12% Bis-Tris precast gel as described before (Sanders et al., 2014). The resulting blot was probed with in-house HIP14 polyclonal antibody (Hayden lab; 1:400) and β-tubulin monoclonal antibody (Sigma; 1:5000). Based on the results of the *Hip14*-ASO dose response time course (Supplementary Figure S1), the 250 nM final concentration for 10 days was used for HIP14 knockdown on wild type and HIP14L-/- mice cortical neuronal cultures.

HIP14L siRNA primers were annealed and inserted into the HindIII/BgIII site of pSuper (Oligoengine) and the pSuper-GFP vector was kindly provided by Dr.Tamoaki Shirao (Gunma University, Japan). Primers used to generate HIP14L siRNA are as follows. Complementary oligonucleotides: 5′-GATCCCCGGAAGCCTTTAAGATCACTTTCAAGAGAAGTG ATCTTAAAGGCTTCCTTTTTA-3′; and 5′-AGCTT AAAAAGGAAGCCTTTAAGATCACTTCTCTTGAAAGTGAT CTTAAAGGCTTCCGGG-3′ (corresponding to the nucleotides 1407 – 1428 of mouse HIP14L mRNA). Primers used to generate scrambled siRNA are as follows: 5′-GATCCCCTTTCGTCCATCTCCAAAGGTTCAAGAGACCTT TGGAGATGGACGAAATTTTTA-3′ and 5′-AGCTTAAAAAT TTCGTCCATCTCCAAAGGTCTCTTGAACCTTTGGAGATG GACGAAAGGG-3′. The specificity of HIP14L siRNA was tested against exogenously expressed HIP14L versus HIP14 in COS-7 cells (Supplementary Figure S2).

### TUNEL (Terminal Deoxynucleotidyl Transferase-Mediated dUTP Nick End Labeling) Assay

At DIV 0 FVB/N mouse striatal neurons were transfected with 2 μg of HIP14L specific siRNA in pSuper-GFP (siRNA) and scrambled siRNA in pSuper-GFP (Control) by electroporation, and then mixed with untransfected cortical neurons at a 1:1 ratio. The DMEM was replaced with Neurobasal medium after 3 h of transfection. At DIV 14, neurons were treated with 50 μM NMDA for 15 min, and then further incubated in conditioned Neurobasal medium for 1 h. Cells were fixed with 4% paraformaldehyde in PBS (pH 7.4) for 20 min and then in 100% methanol at -20°C for 5 min. Cells were stained with GFP antibody for 1 h, followed by incubation with a secondary antibody conjugated to Alexa 488 fluorophore for 1 h at room temperature. Neurons were stained with the ApopTag Red In Situ Apoptosis Detection Kit (Millipore) according to the manufacturer’s instructions. Neurons transfected with various siRNA constructs were counted under the microscope by an observer blinded to the identity of the samples. The number of TUNEL-positive (red) neurons was determined as a fraction of DAPI-positive (blue) in each transfected cell (green). TUNEL-negative background was determined by staining only with secondary Rhodamine antibody. Experiments were repeated in five different culture batches, and 100–200 cells were counted in each experiment. The fractions of TUNEL-positive nuclei determined for each experiment were averaged, and the results are presented as means ± SD.

### Statistics

Figures, tables, and statistical analyses were generated using Microsoft Excel, ImageJ, Prism, Adobe Photoshop or Adobe Illustrator software. Data or bar graphs are presented as the mean ± SD. Significant differences were determined using Prism software (Graphpad, Inc.); direct comparisons were made by the unpaired or paired, two-tail Student’s *t*-test (2-tailed, Mann–Whitney *U*), one-way ANOVA and two-way ANOVA test as appropriate.

## Results

### Palmitoylation of GluN2B Is Reduced in the Striatum of YAC128 Mice by 1 Month of Age

It has been shown that palmitoylation levels of Htt and certain synaptic proteins, including GluA1, PSD-95, SNAP-25 and GAD-65, are significantly reduced in whole brain lysates from YAC128 mice and *Hdh*+/- mice that lack one allele of the HTT gene (Huang et al., 2011; Singaraja et al., 2011; Wan et al., 2013). Reduced palmitoylation has been reported to alter surface membrane trafficking and subcellular localization of these proteins (Huang et al., 2004; Rush et al., 2012; Parsons et al., 2014). Therefore, we first investigated whether palmitoylation of NMDARs is altered in YAC128 mice at an early stage, prior to onset of motor and cognitive deficits.

Striatal and cortical tissues from 1 to 3 month-old FVB/N and YAC128 mice were examined for palmitoylation of GluN2A and GluN2B since 2B-NMDAR (but not 2A-NMDAR) surface expression and subcellular localization is altered soon after birth (20, 22). To compare the palmitoylation levels of GluN2B or GluN2A between experiments, the intensity of the palmitoylation band was normalized to that of the total lysate, using the GluN2B or GluN2A subunit-specific antibodies. Genotype comparisons were made by normalizing these ratios for YAC128 to that of the FVB/N (wild-type, WT) control tissue run on the same gel in the same experiment. Despite variability between experiments indicated by the standard deviation (Figure 1B–E), when comparing between the two genotypes within each experiment (paired genotype ratios are connected by lines), we found a consistent reduction in GluN2B palmitoylation levels in YAC128 striatal tissue at both 1 and 3 months of age (Figure 1A,B); however, we found no genotype difference in cortical tissue at 1 month (Figure 1C), correlating with the increased vulnerability of striatal neurons to degeneration in the earliest stages of HD (Vonsattel and Difiglia, 1998). In contrast, palmitoylation of GluN2A was unchanged in both striatum (Figure 1D) and cortex (Figure 1E) tissues of 1 month-old YAC128 mice, consistent with the lack of change in GluN2A trafficking in YAC128 striatal neurons (Milnerwood et al., 2012).

Reduction of GluN2B palmitoylation in striatal tissue suggested that palmitoylation of GluN2B may undergo palmitoyl cycling, and thereby provide a mechanism to regulate protein trafficking (El-Husseini and Bredt, 2002; Kang et al., 2004, 2008; Huang and El-Husseini, 2005). To determine whether GluN2B palmitoylation is dynamic over a time scale of hours, we examined the turnover rate of palmitate on GluN2B by addition of the PAT inhibitor, 2-bromopalmitate (2-BP, 100 μM), to co-cultures of striatal medium-spiny neurons (MSN) with cortical neurons (CTX) from FVB/N mice for 5 h as described before (Kang et al., 2008). In the presence of 2-BP, significant reduction of GluN2B palmitoylation was observed (Figure 1F). These data, combined with the previously reported 18–24 h half-life of GluN2 NMDAR subunits (Huh and Wenthold, 1999), suggest that GluN2B palmitoylation in striatal neurons is also a dynamic event, consistent with the previous finding that this process is regulated by synaptic activity in cultured cortical neurons (Hayashi et al., 2009). Taken together, our results suggest that the palmitoylation of GluN2B is regulated dynamically and in a tissue-specific manner, and thus may contribute to the early trafficking alteration of GluN2B-type NMDARs in the YAC128 mouse striatum.

### Palmitoylation of Cys Cluster II-Containing GluN2B C-Terminal Fragment Is Significantly Reduced in Striatum of YAC128 Mice

**FIGURE 2:**
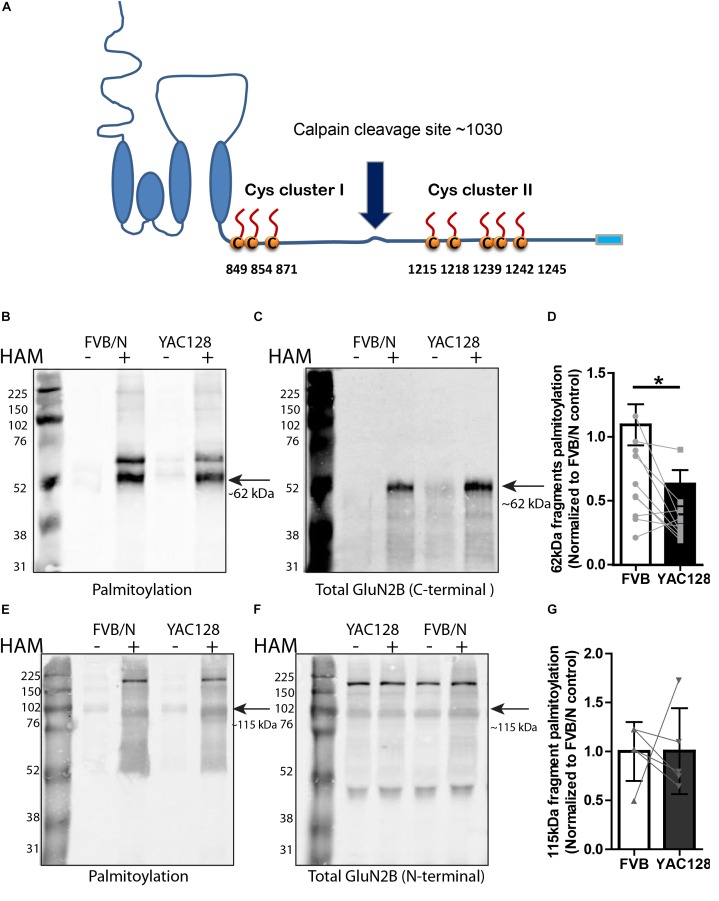
Calpain cleavage of GluN2B to isolate Cys cluster II-containing C-terminal fragment reveals reduced palmitoylation in YAC128 striatum. **(A)** Cartoon indicating relative location of GluN2B Cys clusters I and II and the major calpain cleavage site. Each panel of image data shows results from one representative gel and the dividing line(s) indicate where lanes were removed for ease of comparing genotypes under identical experimental conditions. **(B–D)** Palmitoylation of Cys cluster II-containing C-terminal fragment was examined by IP/Btn-BMCC method. Representative blots show that palmitoylation (detected by probing with streptavidin Alexa 680, **B**) of GluN2B Cys cluster II-containing C-terminal fragment as a ratio to total GluN2B levels (detected by C-terminal specific antibody MA1-2014, as shown in **C**) was significantly decreased in striatum from YAC128 compared to FVB/N wild-type mice. Black arrow = ∼62 kDa cluster II-containing C-terminal fragment. **(D)** Summary graph indicates the corresponding ratio of palmitoylation signal (calculated as in Figure 1) for YAC128 normalized to the FVB/N control. Data presented for striatal tissue from 10 independent experiments; bars represent means ± SD ^∗^*p* < 0.05 (paired *t*-test) and data points from same experiment are connected by lines. **(E–G)** Palmitoylation of Cys cluster I-containing N-terminal fragment was examined by ABE/western blotting method. **(E)** Purified palmitoylated GluN2B was detected with GluN2B N-terminal specific antibody (AGC-003); total GluN2B expression is shown in representative blot in **(F)**, using the same ACG-003 antibody. Black arrow = ∼115 kDa cluster I-containing N-terminal fragment. **(G)** Palmitoylation level of cluster I-containing fragment of GluN2B was calculated as ratio of palmitoylation signal (shown in **E**) to the total protein signal (in **F**); summary graph indicates corresponding palmitoylation level in YAC128 normalized to FVB/N, as in Figure 1. Data presented for striatal tissue from 7 independent experiments; bars represent means ± SD and data points from same experiment are connected by a line.

Since we found reduced palmitoylation of GluN2B in YAC128 striatal tissue, and previous work showed that palmitoylation of GluN2B occurs at two distinct Cys clusters that differentially regulate 2B-NMDAR retention in the Golgi apparatus and surface expression in cortical neurons (Hayashi et al., 2009; Mattison et al., 2012), we asked whether one or both GluN2B Cys clusters show altered palmitoylation in the striatum of YAC128 mice. Previous studies demonstrated that GluN2B is cleaved by calpain at approximately amino acid 1030 in the C-terminal domain, which is between Cys cluster I and Cys cluster II (Simpkins et al., 2003; Cowan et al., 2008), to generate N-terminal 115 kDa and C-terminal 62 kDa fragments of GluN2B (Figure 2A). Therefore, we isolated full-length GluN2B by immunoprecipitation using an N-terminal epitope-specific antibody, followed by *ex vivo* calpain cleavage. Next, we immunoprecipitated the C-terminal fragment from the reaction supernatant using a C-terminal epitope-directed antibody, then forwarded the precipitate to a biotin-BMCC assay as described before (Drisdel and Green, 2004; Huang et al., 2009) to directly measure palmitoylation of the Cys cluster II-containing C-terminal 62 kDa fragment of GluN2B on western blot (see section “Materials and Methods” for details). Palmitoylation of the cluster II-containing C-terminal fragment of GluN2B was detected by probing with the streptavidin Alexa Fluor^®^680 conjugate and total Cys cluster II-containing C-terminal fragment of GluN2B was probed with an anti-mouse C-terminal directed antibody (MA1-2014). Results demonstrated a significant decrease in GluN2B Cys cluster II palmitoylation in YAC128 versus FVB/N striatum (Figure 2B–D). The relatively low intensity band for total C-terminal GluN2B in the HAM- reaction extracts (Figure 2C) was found in some experiments and likely a result of further proteolytic cleavage during the multi-step Btn-BMCC assay. We speculate that the GluN2B C-terminal fragment in the HAM+ lysates was relatively protected from further proteolytic cleavage by the biotin linkages, and/or the earlier transfer to denaturing conditions (low pH during HAM treatment).

As shown by the blots in Figure 2E,F, using an N-terminal epitope-specific GluN2B antibody, the calpain cleavage of full-length GluN2B was incomplete under the conditions used for these experiments. After testing several different calpain concentrations, with and without the 100 μM calpain inhibitor, for varying treatment times to induce *ex vivo* calpain-mediated proteolysis, we chose the conditions used (10 nM calpain, 10 min at RT, with 100 μM calpain inhibitor-1) in order to generate sufficient amounts of the 62 kDa C-terminal fragment without resulting in its loss due to further proteolytic cleavage. Figure 2E–G demonstrate that palmitoylation of the cluster I-containing N-terminal ∼115 kDa fragment, measured by ABE/western blot analysis, was similar in YAC128 versus FVB/N striatum. We conclude that decreased palmitoylation on Cys cluster II but not Cys cluster I of GluN2B mainly contributes to the reduction of GluN2B palmitoylation in the striatum of YAC128 mice.

### Palmitoylation of GluN2B on Two C-Terminal Cysteine Clusters Differentially Regulates Its Surface Expression in Striatal Neurons From FVB/N and YAC128 Mice

Previous studies showed elevated NMDAR current and GluN2B surface expression that is localized to extrasynaptic sites in striatal neurons of 1 month-old YAC128 mice (Li, 2004; Milnerwood and Raymond, 2007; Milnerwood et al., 2010), correlating with our finding that GluN2B Cys cluster II palmitoylation is reduced in the striatum of YAC128 mice as early as 1 month of age. Therefore, it is important to examine whether the palmitoylation deficient mutants of GluN2B on Cys cluster I or cluster II affect the surface expression of 2B-NMDARs in striatal neurons co-cultured with cortical neurons (MSN-CTX) from FVB/N and YAC128 mice, since MSN-CTX co-cultures reproduce the excitatory glutamatergic input from cortical neurons onto striatal MSNs *in vitro* (Kaufman et al., 2012; Milnerwood et al., 2012).

**FIGURE 3:**
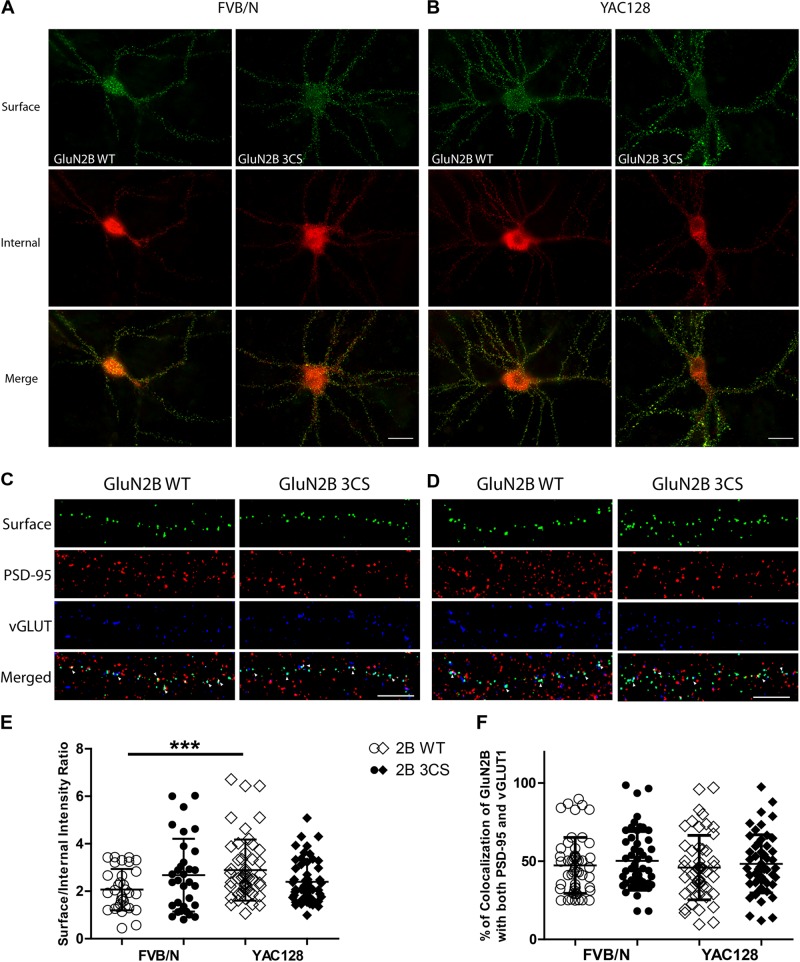
Loss of Cys cluster I (GluN2B 3CS) palmitoylation of GluN2B does not affect the surface expression of 2B-NMDARs in striatal neurons. GFP-tagged GluN2B wild type (WT) and Cys cluster I mutant (GluN2B 3CS) were nucleofected in striatal neurons co-cultured with cortical neurons. **(A)** Images representing the surface/internal expression of GluN2B in striatal neurons in MSN-CTX co-cultures from FVB/N mice. Neuronal cultures were live stained for surface GluN2B (green) with GFP antibody at DIV 18, then fixed and stained for internal GluN2B (red). Merged image shows the total GluN2B expression. Scale bars, 20 μm. **(B)** Representative images of surface (green)/internal (red) expression of GluN2B in striatal neurons co-cultured with cortical neurons from YAC128 mice. **(C,D)** Images representing the colocalization of surface GluN2B (green) with excitatory synaptic markers, PSD-95 (red) and vGLUT1 (blue), in striatal neurons in MSN-CTX co-cultures expressing the wild type and Cys cluster I mutant (GluN2B 3CS). Scale bars, 10 μm. **(E)** Quantitative analysis for the ratio of surface/internal GluN2B intensity. Data from FVB/N and YAC128 co-cultures were acquired in paired experiments (*N* = 4 paired culture batches, 52 cells for each genotype and construct). GluN2B WT surface intensity was significantly enhanced in YAC128 vs. FVB/N striatal neurons (two-way ANOVA, *p* = 0.0310 for mouse genotype, *p* = 0.7749 for GluN2B construct, and *p* = 0.0036 for interaction; ^∗∗∗^*p* < 0.001 by Bonferroni’s *post hoc* test). **(F)** Summary graph of the colocalization of surface puncta of GluN2B with PSD-95 and vGLUT1. Data show no significant difference between genotype or GluN2B construct (two-way ANOVA, *p* = 0.4987 for genotype, *p* = 0.1318 for 3CS mutant, *p* = 0.8475 for interaction; ^n.s^*p* > 0.05 by Bonferroni’s *post hoc* tests). Colocalization data were from 3 independent experiments/38 cells analyzed in FVB/N and 3 independent experiments/44 cells analyzed in YAC128.

To examine whether Cys cluster I palmitoylation is involved in regulating the surface and/or synaptic expression of GluN2B in striatal neurons, GFP-tagged GluN2B WT or GluN2B 3CS (Cys cluster I palmitoylation-deficient mutant) (Hayashi et al., 2009) constructs were nucleofected into striatal neurons of MSN-CTX co-cultures from FVB/N and YAC128 mice at day of plating (DIV 0). Surface expression of GluN2B WT and GluN2B 3CS mutant were detected as green fluorescence and internal subunits as red fluorescence, and quantified as the surface green to internal red fluorescence intensity ratio (see section “Materials and Methods”), as previously described (Milnerwood et al., 2010). Representative patterns of GFP-GluN2B WT and the GFP-GluN2B 3CS mutant in co-cultured striatal neurons are shown in Figure 3A (FVB/N) and Figure 3B (YAC128). Consistent with our previous results (Milnerwood et al., 2012), the surface to internal intensity ratio for GFP-GluN2B WT was significantly higher in striatal neurons from YAC128 mice when compared with FVB/N mice (Figure 3E). In contrast with reduced surface expression of GluN2B 3CS in cortical neurons (Hayashi et al., 2009), we found no significant impact of this mutant on 2B-NMDAR surface expression in striatal neurons from either FVB/N or YAC128 mice (Figure 3E). One possible explanation is that GluN2B 3CS may alter the puncta size of GluN2B or density of GluN2B-containing NMDARs in co-cultured striatal neurons; however, we did not observe alteration of either puncta size or density of GluN2B in striatal neurons (data not shown). Moreover, our results showed that surface-expressed GFP-GluN2B 3CS colocalization with postsynaptic marker PSD-95 and presynaptic marker vGLUT1 was similar to that of GFP-GluN2B WT in striatal neurons from both FVB/N and YAC128 mice (Figure 3C,D,F), indicating that the palmitoylation of GluN2B Cys cluster I does not affect synaptic localization of 2B-NMDARs in striatal neurons.

**FIGURE 4:**
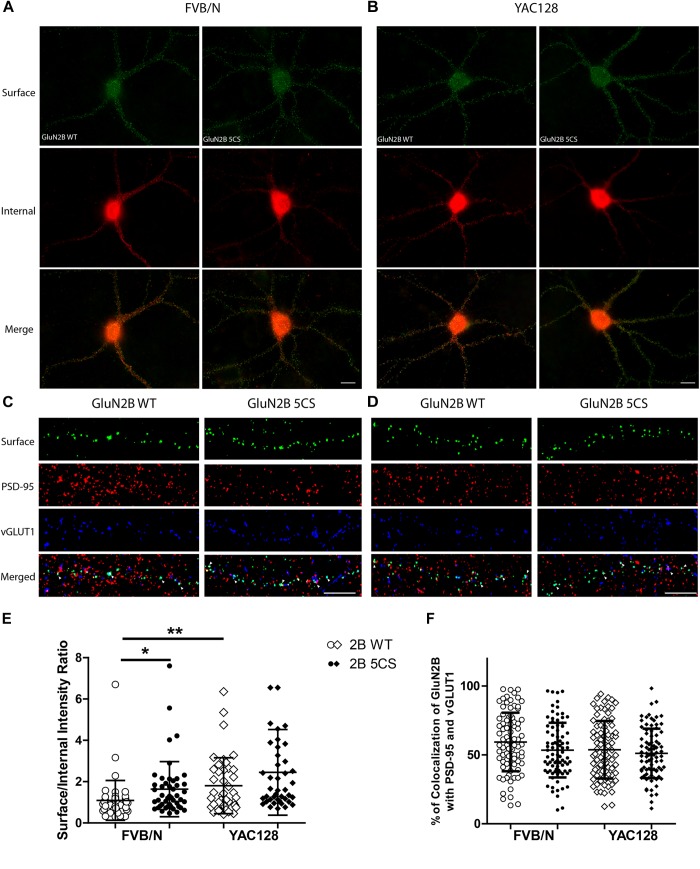
Loss of Cys cluster II (GluN2B 5CS) palmitoylation of GluN2B regulates the surface expression of GluN2B in striatal neurons. GFP-tagged GluN2B wild type (WT) and Cys cluster II mutant (GluN2B 5CS) were nucleofected in striatal neurons co-cultured with cortical neurons. **(A)** Images representing the surface (green)/internal (red) expression of GluN2B in striatal neurons in MSN-CTX co-cultures from FVB/N mice. Merged image shows the total GluN2B expression. Scale bars, 20 μm. **(B)** Representative images of surface (green)/internal (red) expression of GluN2B in striatal neurons co-cultured with cortical neurons from YAC128 mice. **(C,D)** Representative images of surface GluN2B puncta colocalization with the excitatory synaptic markers PSD-95 and vGLUT1 for GluN2B WT and 5CS in FVB/N and YAC128 DIV 18 striatal neurons in MSN-CTX co-cultures. Scale bars, 10 μm. **(E)** Quantitative analysis for the ratio of surface to internal GluN2B intensity was performed for data from paired experiments from 4 batches each of FVB/N and YAC128 MSN-CTX co-cultures. Similar to results shown in Figure 3E, GluN2B WT surface intensity was significantly enhanced in YAC128 vs. FVB/N striatal neurons; as well, surface intensity was significantly enhanced in FVB/N but not YAC128 striatal neurons expressing the GluN2B 5CS [FVB/N: *N* = 4(48 cells), YAC128: *N* = 4(42 cells)] when compared to neurons expressing GluN2B WT [FVB/N: *N* = 4(48 cells), YAC128: *N* = 4(42 cells)]. Significant by two-way ANOVA, *p* = 0.0012 for genotype, *p* = 0.0129 for 2B construct, and *p* = 0.9970 for interaction; ^∗∗^*p* < 0.01, ^∗^*p* < 0.05 by Bonferroni’s *post hoc* test. **(F)** Summary graph shows similar colocalization of GluN2B WT and 5CS punctae with both PSD-95 and vGLUT1. Colocalization data were from 4 independent experiments/38 cells analyzed in FVB/N and 4 independent experiments/36 cells analyzed in YAC128. Two-way ANOVA, *p* = 0.1785 for genotype, *p* = 0.0592 for 5CS mutant, *p* = 0.1571 for interaction; *p* > 0.05 by Bonferroni’s *post hoc* test.

Next, to assess whether reduced palmitoylation of GluN2B Cys cluster II affects surface and/or synaptic localization in striatal neurons, we compared the surface to internal intensity ratio between GFP-GluN2B WT and GFP-GluN2B 5CS (Cys cluster II palmitoylation-deficient mutant). We found the surface to internal intensity ratio significantly increased for GluN2B 5CS when compared with GluN2B WT in FVB/N but not in YAC128 striatal neurons (Figure 4A,B,E). Notably, the expression of GluN2B 5CS in FVB/N striatal neurons increased 2B-NMDAR surface expression to levels similar to GluN2B WT in YAC128 striatal neurons (Figure 4E). In addition, the increased GluN2B surface expression was not colocalized with synaptic PSD-95 and vGLUT1 (Figure 4C,D,F), suggesting that reduced palmitoylation of GluN2B Cys cluster II promotes surface expression of 2B-NMDARs at extrasynaptic sites, which was seen in cortical neurons in a previous study (Mattison et al., 2012).

### Huntingtin-Interacting Protein 14 (HIP14) and HIP14L Differentially Modulate Palmitoylation of GluN2B Cys Clusters I and II

The differential effect of palmitoylation on two GluN2B Cys clusters on 2B-NMDAR surface expression in striatal and cortical neurons raises the question as to whether GluN2B palmitoylation could be differentially regulated in FVB/N and YAC128 mice. Previous studies showed that HIP14 and HIP14L are the only two PATs that palmitoylate and interact with the Htt protein (Singaraja et al., 2002; Huang et al., 2011). HIP14 activity and palmitoylation levels are significantly reduced in brains of YAC128 mice (Singaraja et al., 2011), In addition, *Hip14*-/- mice recapitulate neuropathological features of HD but is an earlier, developmental phenotype (Singaraja et al., 2011), whereas *Hip14L*-/- mice reproduce the age-onset, progressive neuropathological features of HD (Sutton et al., 2013); both show reduced interaction with mHtt. We therefore investigated whether HIP14 and HIP14L can act as PATs for GluN2B and differentially regulate GluN2B palmitoylation on the two Cys clusters.

**FIGURE 5:**
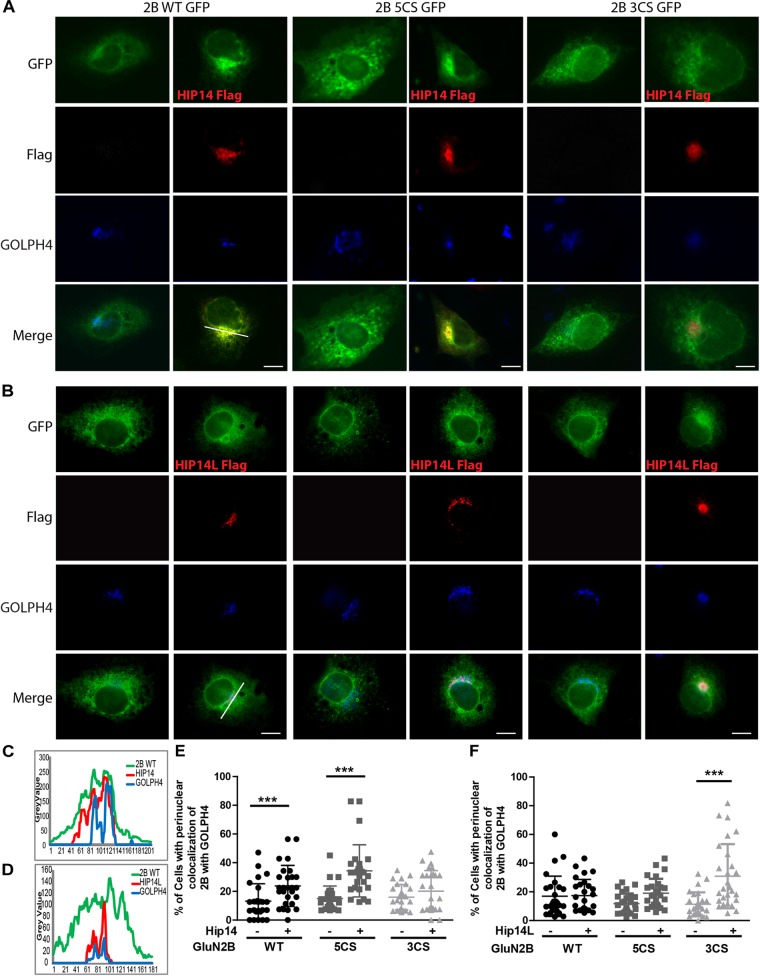
HIP14 and HIP14L differentially modulate trafficking of GluN2B in COS-7 cells. Flag-tagged HIP14 or HIP14L construct, together with GFP-tagged GluN2B WT, GluN2B 5CS or GluN2B 3CS, and HA-tagged GluN1-1A, were transfected into COS-7 cells. 2B-NMDAR and/or HIP14/HIP14L subcellular distribution was assessed by immunostaining with anti-GFP (green channel, GluN2B), anti-Flag (red channel, HIP14 or HIP14L) and *cis*- and medial-Golgi marker, GOLPH4 (blue channel) 36 h after transfection. **(A)** GFP-tagged GluN2B alone localizes to the cytoplasm in a diffuse pattern. Exogenous HIP14 promotes the accumulation of GluN2B WT and GluN2B 5CS into the perinuclear region (indicated by GOLPH4). **(B)** Exogenous HIP14L preferentially promotes the accumulation of GluN2B 3CS into the perinuclear region. Scale bars, 20 μm. **(C)** Examples of line scan profiles in perinuclear region for GluN2B WT-GFP cotransfected with HIP14-Flag. **(D)** Examples of line scan profiles in perinuclear region for GluN2B WT-GFP co-transfected with HIP14L-Flag. **(E)** Line scan analysis for perinuclear region distribution was obtained from 30 cells/condition with or without HIP14 co-transfection. Consistent with images, HIP14 overexpression increases the percentage of cells showing perinuclear colocalization of GluN2B WT and 5CS with GOLPH4. Data was obtained from 3 independent experiments and bars represent means ± SD. Two-way ANOVA, *p* = 0.013 for interaction, *p* = 0.016 for mutant, ^∗∗∗^*p* < 0.0001 by Bonferroni *post hoc* tests. **(F)** Line scan analysis for perinuclear region distribution was obtained from 30 cells/condition with or without HIP14L co-transfection. HIP14L overexpression enhances the percentage of cells showing perinuclear colocalization of GluN2B 3CS with GOLPH4 in perinuclear regions. Data was obtained from 3 independent experiments and bars represent means ± SD. Two-way ANOVA, *p* = 0.001 for interaction, *p* = 0.0728 for mutant, ^∗∗∗^*p* < 0.0001 by Bonferroni *post hoc* tests.

**FIGURE 6:**
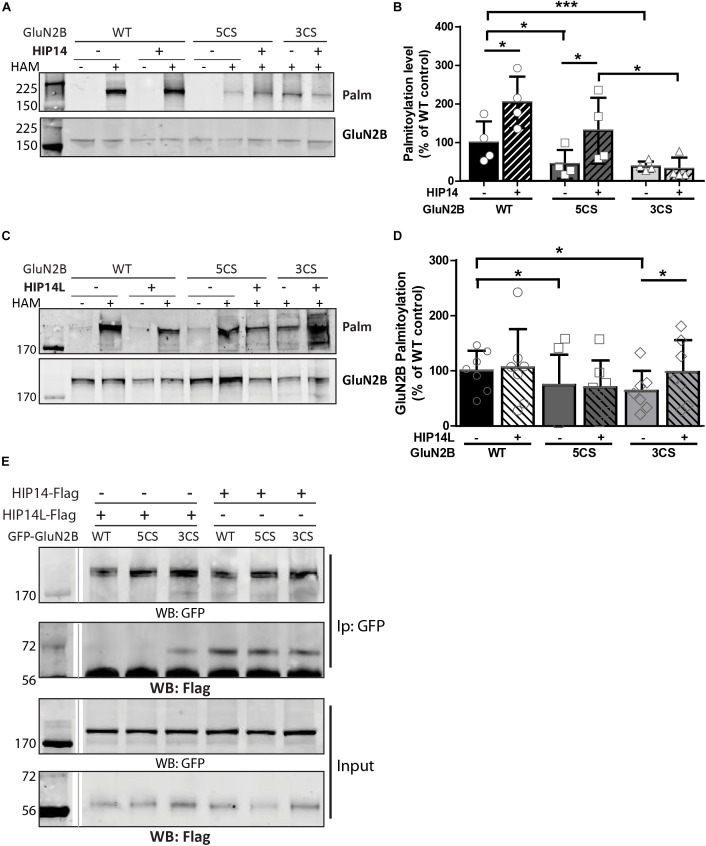
Huntingtin-interacting protein 14 (HIP14) and HIP14L differentially modulate palmitoylation of GluN2B on Cys clusters I and II. GFP-tagged GluN2B WT, GluN2B 5CS and GluN2B 3CS constructs together with HA-tagged GluN1-1A, were transfected in COS cells with or without HIP14-Flag or HIP14L-Flag for 36 h, and GluN2B palmitoylation level changes were detected by ABE assay/western analysis. Each of the representative western blot panels is an example from one blot; the dividing line in **(E)** indicates where some lanes on the original blot were removed. **(A)** Representative western blots show that HIP14 significantly enhanced the palmitoylation of GluN2B WT and GluN2B 5CS, but not GluN2B 3CS. **(B)** Graph summarizing the quantitative analysis for GluN2B palmitoylation with HIP14-Flag co-transfection. The value indicates the percentage of GluN2B antibody intensity where 100% refers to the GluN2B WT control without HIP14. Data is presented from 4 independent experiments as mean ± SD (Two-way ANOVA, *p* = 0.0114 for HIP14, *p* = 0.0014 for mutant GluN2B, *p* = 0.1127 for interaction; ^∗^*p* < 0.05, ^∗∗∗^*p* < 0.001 by Bonferroni *post hoc* tests). **(C)** Representative western blots show that HIP14L enhanced the palmitoylation of GluN2B 3CS but not GluN2B WT or 5CS. **(D)** Graph summarizing the quantitative analysis from 7 independent experiments (Two-way ANOVA, *p* = 0.7158 for HIP14L, *p* = 0.0959 for mutant GluN2B, *p* = 0.8228 for interaction). Notably, each of the mutant GluN2B constructs showed reduced palmitoylation compared to GluN2B WT in absence of HIP14 **(B)** or HIP14L **(D)** by paired *t*-test (^∗^*p* < 0.05), and the palmitoylation of GluN2B 3CS was increased back to the GluN2B WT level with HIP14L co-transfection in COS-7 cells (paired *t-test, p* = 0.0343 for 3CS mutant with/without HIP14L). **(E)** GFP-GluN2B (WT, 5CS, and 3CS) together with HA-GluN1-1A constructs were transfected with either HIP14-Flag or HIP14L-Flag in COS-7 cells; cells were lysed after 36 h and subjected to co-immunoprecipitation with GFP antibody. The interactions were detected with Flag antibody by western blot. Interaction between GluN2B and HIP14L requires the presence of Cluster II cysteines; in contrast, the association of GluN2B with HIP14 is observed in the absence of either one of the GluN2B Cys clusters.

Three common approaches to identify putative substrates of a specific PAT are to: (i) use immunocytochemistry to compare putative substrate subcellular distribution alone and with co-transfection of PAT constructs in heterologous cells, looking for substrate accumulation in the perinuclear region as a proxy for increased substrate palmitoylation; (ii) examine substrate palmitoylation level changes by biochemical palmitoylation assay after transfection of the putative substrate alone or together with its potential PAT in heterologous cells; and (iii) identify an interaction between a PAT and its putative substrate by co-immunoprecipitation. First, we expressed Flag-tagged HIP14 or HIP14L with either GFP-tagged GluN2B WT, GluN2B 5CS or GluN2B 3CS, together with HA-tagged GluN1-1A, in COS-7 cells. GluN2B subcellular distribution was assessed 36 h after transfection. Since both HIP14 and HIP14L are predominantly localized in the Golgi compartment (Huang et al., 2004; Sutton et al., 2013), we stained with the *cis*- and medial-Golgi marker, GOLPH4. Here, we found that HIP14 significantly enhanced the perinuclear accumulation of GluN2B WT and GluN2B 5CS but not GluN2B 3CS (Figure 5A,C,E), suggesting that HIP14 can act as a PAT for GluN2B WT, mainly on Cys cluster I but not Cys cluster II in this overexpression system. In contrast, HIP14L did not affect GluN2B WT or 5CS subcellular distribution but significantly enhanced the perinuclear accumulation of GluN2B 3CS in COS-7 cells, suggesting palmitoylation of cluster II by HIP14L is modulated by palmitoylation on cluster I (Figure 5B,D,F). Next, we directly measured GluN2B palmitoylation by ABE assay after transfection with or without HIP14 or HIP14L in COS-7 cells for 36 h. We found that mutation of GluN2B at either Cys cluster I (GluN2B 3CS) or Cys cluster II (GluN2B 5CS) decreased GluN2B palmitoylation in comparison with GluN2B WT in the absence of HIP14 or HIP14L overexpression (Figure 6A–D). Further, in agreement with the imaging data in Figure 5, palmitoylation of GluN2B WT and GluN2B 5CS, but not GluN2B 3CS, was enhanced by co-expression of HIP14 (Figure 6A,B), indicating that HIP14 is a PAT for GluN2B cluster I in COS-7 cells. In contrast, only palmitoylation of GluN2B Cys cluster II (GluN2B 3CS) was enhanced in the presence of HIP14L in COS cells (Figure 6C), and this enhancement brought GluN2B 3CS palmitoylation levels back to those of wild type GluN2B (Figure 6D). Taken together, these results point to the possibility of differential regulation of GluN2B trafficking by HIP14 and HIP14L.

We next addressed whether differential palmitoylation of the two GluN2B Cys clusters by two phylogenetically related PATs (HIP14 and HIP14L) correlates with differential interaction between these PATs and their substrates. Previous studies suggest the interaction between PATs and their specific substrates may be transient, during the enzymatic transfer of palmitate to the substrates (Huang et al., 2009). We examined interaction of GluN2B with HIP14 and HIP14L by co-immunoprecipitation after transfection in COS-7 cells for 36 h. HIP14 was found to interact with GluN2B WT (both Cys clusters available), GluN2B 5CS (Cys cluster I only) and GluN2B 3CS (Cys cluster II only) in COS cells (Figure 6E). Notably, although the interaction between HIP14 and GluN2B Cys cluster II was detected by co-immunoprecipitation, no enhancement of GluN2B Cys cluster II palmitoylation by HIP14 was detected (Figure 6A), providing evidence to support the idea that HIP14 acts as a PAT for GluN2B Cys cluster I. In contrast, HIP14L showed negligible co-immunoprecipitation with GluN2B WT or 5CS (Cys cluster I only), but showed weak association with GluN2B 3CS (Cys cluster II only) (Figure 6E), consistent with the data in Figure 6C,D and supporting the idea that HIP14L is able to interact with and palmitoylate Cys cluster II of GluN2B when Cys cluster I palmitoylation is reduced/absent. These data and results of imaging and western blot for GluN2B palmitoylation in COS cells are summarized in Table 1.

**Table 1:** Summary of changes of wild type and two palmitoylation deficiency mutant of GluN2B in the presence of HIP14 and HIP14L in COS cells.

	GluN2B WT (full length)		GluN2B 5CS (cluster 1)		GluN2B 3CS (cluster II)	
			
	HIP14	HIP14L	HIP14	HIP14L	HIP14	HIP14L
Perinuclear accumulation	+++	–	+++	-	-	+++
Palmitoylation assay	+++	–	+++	-	-	+++
PAT-substrate interaction	++	+/-	+	-	+	+


### Palmitoylation of GluN2B in Striatal Neurons Is Decreased in HIP14L-Deficient Mice but Not by Knock-Down of HIP14

**FIGURE 7:**
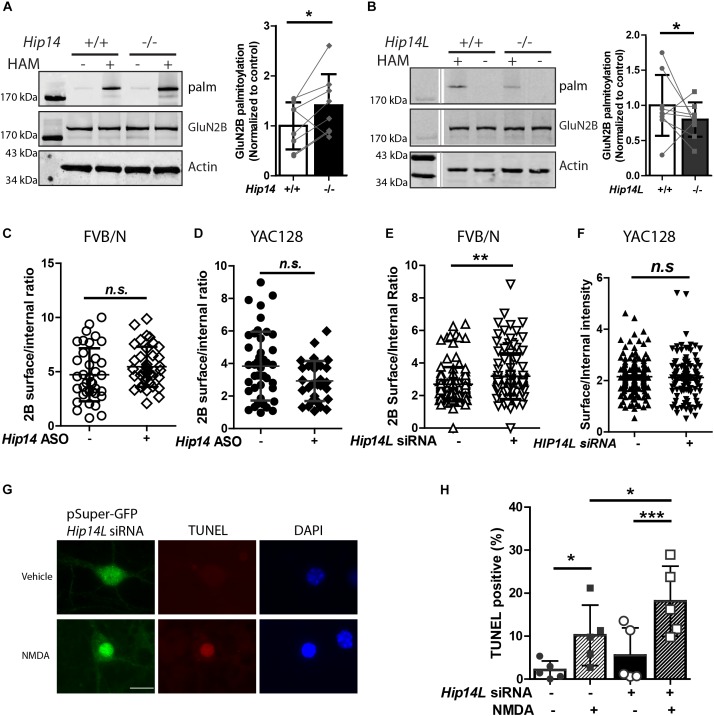
HIP14L deficiency reduces GluN2B palmitoylation and increases 2B-NMDAR surface expression and NMDA-induced cell death in striatal neurons. Each of the representative western blot panels is an example from one blot; the dividing line in **(B)** indicates where some lanes on the original blot were removed. **(A)** GluN2B palmitoylation was significantly increased (41.2 ± 5.32%) in striatum from *Hip14*–/– mice vs. *Hip14*+/+ control. Data are presented from 8 independent experiments in which tissue from HIP14–/– and wild-type control were paired; lines connect the points from paired data. Bars represent means ± SD ^∗^*p* < 0.05 (Paired *t*-test, *p* = 0.0411). **(B)** GluN2B palmitoylation was significantly decreased in striatum from *Hip14L*–/– mice (19.1 ± 1.05%) when compared with *Hip14L*+/+ control. Data are presented from 9 independent experiments, as described in **(A)**. ^∗^*p* < 0.05 (Paired *t*-test, *p* = 0.0336). **(C)** The surface expression of 2B-NMDARs in striatal neurons of MSN-CTX co-cultures from FVB/N mice showed a trend toward an increase with 250 nM *Hip14* ASO treatment for 10 days (*N* = 6/38 cells; n.s. *p* = 0.0676, Non-parametric test, Mann–Whitney *U*). Representative images are shown in Supplementary Figure S3A. **(D)** A trend toward decreased surface 2B-NMDARs was observed in MSN-CTX co-cultured striatal neurons from YAC128 mice with 250 nM *Hip14* ASO treatment for 10 days (*N* = 5/42 cells; ^n.s^*p* = 0.0770, Non-parametric test, Mann–Whitney *U*). However, these trends did not reach statistical significance for either genotype. Representative images are shown in Supplementary Figure S3B. **(E,F)** The surface expression of GFP-GluN2B measured at DIV 18 in striatal neurons of MSN-CTX co-cultures from FVB/N mice was significantly increased (22.6 ± 5.72%) when GFP-GluN2B WT was co-transfected with pSuper-*Hip14L* siRNA (*N* = 5/51 cells; ^∗∗^*p* = 0.0058, non-parametric test, Mann–Whitney *U*). However, knockdown of HIP14L had no effect on surface expression of GFP-GluN2B in striatal neurons from YAC128 MSN-CTX co-cultures. Representative images are shown in Supplementary Figures 3C,D. **(G,H)** Neurons were transfected with pSuper-GFP *Hip14L* siRNA (siRNA+) or pSuper-GFP Scrambled siRNA (control–), treated at DIV 14 with vehicle or 50 μM NMDA for 15 min, returned to conditioned medium at 37°C for 1 h, then fixed and stained with GFP antibody (green, siRNA construct), red stain for TUNEL-positive neurons and DAPI (blue) to identify nuclei. **(G)** Representative images from cells transfected with pSuper-GFP *Hip14L* siRNA show a typical TUNEL-positive neuron in the bottom panel (NMDA treated), in which the nucleus is condensed and homogeneously stained with DAPI, and the red TUNEL stain fills the nucleus. **(H)** The percentage of TUNEL-positive cells among all siRNA-transfected (green) neurons (representing apoptotic cell death) after NMDA treatment was significantly increased upon knockdown of HIP14L in striatal neurons of MSN-CTX co-cultures from FVB/N mice. Experiments were repeated five times and 100–200 cells in each experiment were counted. The results are presented as mean ± SD (one-way ANOVA, ^∗^*p* < 0.05, ^∗∗∗^*p* < 0.001, Bonferroni’s multiple comparison test).

While we found that HIP14 palmitoylates GluN2B on Cys cluster I and HIP14L can palmitoylate GluN2B on Cys cluster II in transfected COS-7 cells, whether loss of HIP14 or HIP14L affect GluN2B palmitoylation in the brain remained a question. First, we examined the palmitoylation of GluN2B in *Hip14*-/- mouse striatum (Figure 7A). Surprisingly GluN2B palmitoylation was not decreased, but rather increased (41.2% ± 3.3%) in *Hip14*-/- striatal tissue, suggesting that GluN2B palmitoylation by HIP14L and/or other PATs was facilitated as a result of 90% loss of HIP14 (these gene-trap knock-out mice retain 10% HIP14 expression) (Young et al., 2012). Consistent with this, previous studies had shown that palmitoylation of Htt, another major substrate of HIP14, was not decreased in *Hip14*-/- mice brain (Singaraja et al., 2011), possibly due to compensation by HIP14L (Singaraja et al., 2011; Sutton et al., 2013; Sanders et al., 2014). Next, we found that GluN2B palmitoylation was significantly reduced by 19.1% ± 1.1% in the striatum of mice with complete knock-out of HIP14L (*Hip14L*-/-) (Figure 7B), in contrast with the lack of change in Htt palmitoylation in the *Hip14L*-/- mouse brain (Sutton et al., 2013).

### Effect of HIP14 or HIP14L Knock-Down on 2B-NMDAR Surface Expression and Susceptibility to NMDA Toxicity in Striatal Neurons in MSN-CTX Co-culture

To investigate the effect of the two huntingtin-associated PATs, HIP14, and HIP14L, on 2B-NMDAR trafficking in striatal neurons, we used MSN-CTX co-cultures in which GFP-tagged GluN2B was transfected selectively into striatal MSNs. First, we tested the effect of HIP14 knock-down by treating FVB/N and YAC128 CTX-MSN co-cultures with/without *Hip14* ASO (Supplementary Figure S1) and measured surface to internal ratio of GFP-tagged GluN2B. Results showed no significant effect of *Hip14* ASO treatment on 2B-NMDAR surface/internal ratio in either FVB/N (Figure 7C) or YAC128 (Figure 7D) MSNs. These results are consistent with unaltered surface expression results from the GluN2B 3CS mutant in CTX-MSN co-cultures. Together with the lack of effect of loss of HIP14 on GluN2B palmitoylation in mouse striatal tissue, these data suggest that although other PATs may compensate for loss of HIP14 palmitoylation on Cluster I, that site does not play a significant role in regulating 2B-NMDAR trafficking in striatal neurons.

Next, to determine whether endogenous HIP14L is important for palmitoylation-dependent trafficking of GluN2B, we generated a small interference RNA (siRNA) that specifically disrupted *Hip14L* expression (Supplementary Figure S2), and examined whether disruption of HIP14L expression altered surface trafficking of GluN2B. As expected, striatal neurons transfected with *Hip14L* siRNA showed a significant increase (22.6% ± 1.72%) in the surface/internal ratio of GluN2B in FVB/N (Figure 7E) but not in YAC128 co-cultures (Figure 7F). Taken together, these results suggest that unlike GluN2B palmitoylation on Cluster I by HIP14, Cluster II palmitoylation by HIP14L contributes to GluN2B-containing NMDAR trafficking in striatal neurons.

An important question is whether altered GluN2B trafficking by defective GluN2B palmitoylation in striatal neurons influences cell death. Since loss of HIP14 had no effect on 2B-NMDAR trafficking, we focused on HIP14L for these experiments. We asked whether the increased 2B-NMDAR surface expression found with HIP14L knockdown in FVB/N striatal neurons was associated with enhanced excitotoxic cell death. The pSuper-GFP-*Hip14L* siRNA (“+”) and pSuper-GFP-scrambled siRNA (control, “-”) were transfected at DIV 0 into striatal neurons co-cultured with untransfected cortical neurons from FVB/N mice; NMDA-induced cell death was assessed by using the TUNEL assay at DIV 14. We found that decreasing HIP14L expression in FVB/N striatal neurons increased their susceptibility to NMDA treatment (Figure 7G,H); after subtracting background cell death in the absence of NMDA, the NMDA-induced cell death in cultures treated with scrambled siRNA was 8.1 ± 5.3% whereas it was 12.6 ± 3.6% in cultures treated with HIP14L siRNA, suggesting that reduced palmitoylation of Cluster II is detrimental to neuronal viability. These results establish that HIP14L is critical for regulating surface expression of GluN2B and neuronal viability in MSN-CTX co-cultures.

## Discussion

**FIGURE 8:**
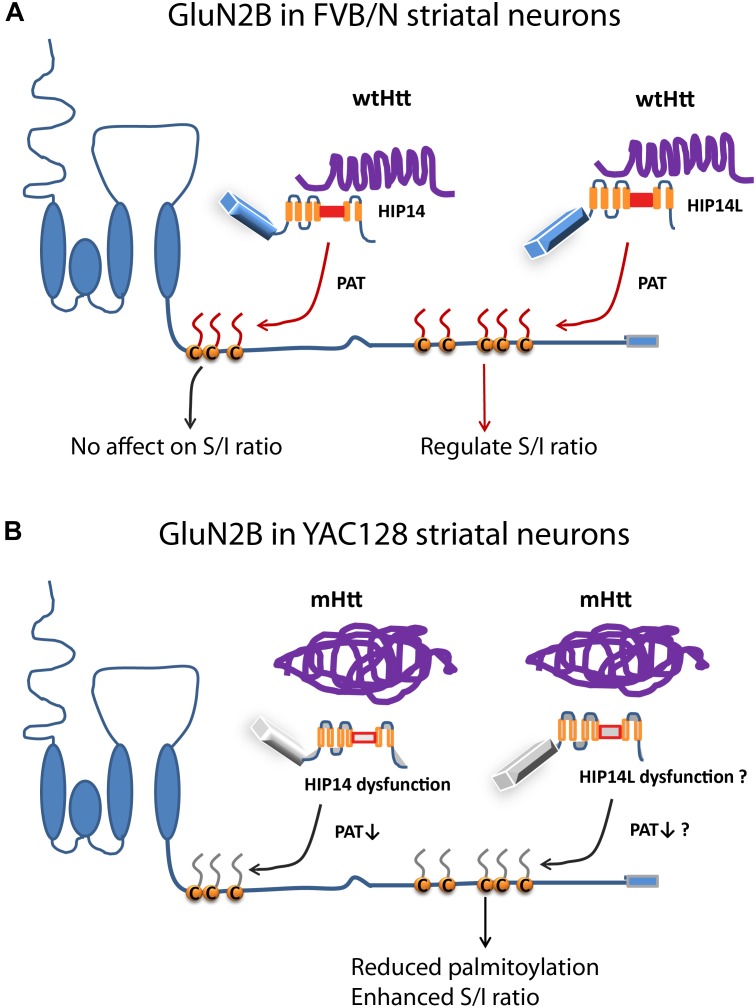
Differential regulation of palmitoylation on two GluN2B Cys clusters by HIP14 and HIP14L. Cartoon diagram of GluN2B structure and modulation by palmitoylation on clusters I and II. **(A)** In FVB/N striatal neurons, wild-type Htt associates with HIP14 and acts as cofactor to promote HIP14 PAT activity, which can palmitoylate GluN2B Cys cluster I. Wild-type Htt also associates with HIP14L, which can palmitoylate cluster II when cluster I palmitoylation is reduced. Palmitoylation of Cys cluster II but not Cys cluster I regulates GluN2B surface localization in FVB/N striatal neurons. **(B)** In YAC128 striatal neurons, aggregated mHtt protein decreases its association with HIP14, leading to reduced palmitoylation and PAT enzyme activity, which should impact GluN2B Cys cluster I palmitoylation; however, it is possible that Cys cluster I palmitoylation is compensated by other PATs. As well, aggregated mHtt protein decreases its association with HIP14L, which may contribute to reduced palmitoylation of GluN2B on Cys cluster II and increased surface expression of GluN2B-containing NMDARs. S/I Ratio (surface/internal ratio); 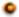 Cysteine; 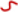 palmitate; 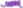 wtHtt; 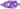 mHtt; 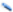 Ankyrin repeat domain (ARD); 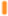 transmembrane domain (TMD); 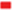 DHHC domain; 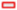 DHHC dysfunction.

This is the first study, to our knowledge, to show that: (i) altered palmitoylation on cluster II of GluN2B regulates extrasynaptic surface expression in striatal neurons; (ii) the increase in GluN2B-containing NMDAR extrasynaptic localization and function previously found in a variety of HD mouse models (Milnerwood et al., 2010; Botelho et al., 2014; Plotkin et al., 2014) may be linked to a reduction in palmitoylation on cluster II of GluN2B (Figure 1, 2, 4). Moreover, the latter finding may be explained by altered function of the huntingtin-interacting protein, HIP14L (zDHHC13), which we found to be a PAT for cluster II in GluN2B (Figure 5, 6), as demonstrated by the fact that reduced function of HIP14L in striatal neurons results in decreased GluN2B palmitoylation, increased 2B-NMDAR surface expression, and also increased neuronal susceptibility to NMDA-induced apoptosis (Figure 7). Enhanced NMDA excitotoxicity is an early stage feature of many full-length HD mouse models and has been linked to elevated expression and activation of 2B-containing extrasynaptic NMDARs (Okamoto et al., 2009; Milnerwood et al., 2010, 2012; Dau et al., 2014), an association that has been documented in other neurological diseases as well (Hardingham and Bading, 2010; Parsons and Raymond, 2014). Together, these results strongly suggest a link between mutant Htt and early enhanced excitotoxic vulnerability of striatal neurons in HD through altered GluN2B palmitoylation by HIP14L, one of two unique Huntingtin-interacting PATs, that differentially interact with and modulate the palmitoylation of GluN2B on the two Cys clusters (Figure 8).

It is critical to uncover mechanisms of 2B-NMDAR mis-trafficking in HD, because most HD mouse models show enhanced NMDA current (Raymond et al., 2011). Moreover, GluN2B is a hub protein for the postsynaptic Htt interactome (Shirasaki et al., 2012), and several lines of evidence support the idea of altered NMDAR signaling in human HD striatum. A disproportionate loss of NMDAR binding in putamen from early HD cases suggested that excessive NMDAR activation had resulted in preferential loss of neurons expressing high levels of these receptors (Young et al., 1988). Before the HD gene was discovered, it was determined that striatal injection of NMDAR agonists resulted in selective loss of striatal MSNs, along with behavioral features of HD, in both rodents and non-human primates (Beal et al., 1991; Fan and Raymond, 2007). Gene association studies in HD patient cohorts have identified variants in the *GRIN2B* gene as contributing to variability in age of onset (Arning et al., 2005, 2007). Moreover, there is evidence of attenuated NMDAR-dependent synaptic plasticity in humans with prodromal HD as assessed by transcranial magnetic stimulation and using motor output as the read-out (Huang et al., 2007; Orth et al., 2010). Here we have demonstrated reduced palmitoylation of GluN2B on Cys cluster II in striatal neurons in YAC128 HD mice, and our data using GluN2B palmitoylation-resistant mutants in cultured striatal MSNs strongly suggest this mechanism contributes to accelerated forward trafficking and enhanced extrasynaptic surface expression of 2B-NMDAR in HD. Unlike GluN2B, the palmitoylation of GluN2A was not affected in early stage striatum (and cortex) in YAC128 HD mice, consistent with the lack of change in 2A-NMDAR trafficking in YAC128 striatal MSNs (Milnerwood et al., 2012).

Our data revealed that palmitoylation of GluN2B is reduced in the striatum but not cortex of one month-old YAC128 HD mice, correlating with the early increase in extrasynaptic 2B-NMDARs in striatal neurons (Milnerwood et al., 2010). Moreover, palmitoylation is specifically reduced on GluN2B Cys cluster II. Furthermore, we observed that eliminating palmitoylation on GluN2B Cys cluster II (GluN2B 5CS) in FVB/N wild-type striatal neurons in MSN-CTX co-cultures significantly increased 2B-NMDAR surface to internal ratio to match that of YAC128 MSNs. Moreover, the increased surface expression of GluN2B 5CS-containing NMDARs was not colocalized with synaptic PSD95 and vGLUT1, indicating a specific increase in extrasynaptic surface receptors. Taken together, these results support the idea that reduced palmitoylation on GluN2B cluster II contributes to accelerated forward trafficking and enhanced distribution of 2B-NMDAR to extrasynaptic sites in YAC128 striatal neurons (Fan et al., 2007; Milnerwood et al., 2010, 2012; Parsons and Raymond, 2014). It remains unknown why this reduction in GluN2B palmitoylation occurs selectively in striatal neurons at this early disease stage; however, we speculate that the relatively low HIP14L mRNA expression in striatum vs. cortex found in FVB/N mice (Sutton et al., 2013) renders striatal neurons more susceptible to reduction in HIP14L function in the presence of mHtt expression.

In contrast with results from GluN2B 5CS-containing NMDARs, we found that absence of palmitoylation on GluN2B Cys cluster I (GluN2B 3CS) did not affect surface expression or synaptic localization of 2B-NMDARs in striatal neurons from either FVB/N wild-type or YAC128 mice. Our results in striatal neurons differ from a previous study in cortical neurons, which showed reduced synaptic NMDAR expression for the 3CS mutant (Hayashi et al., 2009). Further studies will be required to investigate distinct signaling pathways and interacting partners in cortical and striatal neurons.

Theoretically, the number of palmitoylated cysteines present in a protein should not affect the ABE assay, such that one palmitoylated cysteine should produce the same result as larger numbers in terms of bringing down the individual GluN2B subunits. However, experimentally we found that the ABE assay is sensitive to the numbers of palmitoylated cysteines on individual GluN2B subunits. The data shown in Figure 1A,B, 2 indicate that ABE detects a reduction in palmitoylation on GluN2B cluster II when cluster I palmitoylation is intact in full-length GluN2B. Moreover, when we compared COS cells expressing wild-type GluN2B to cells expressing either GluN2B-3CS (cluster I cannot be palmitoylated) or GluN2B-5CS (cluster II cannot be palmitoylated) in side-by-side experiments, we found a reduction of ∼50% in palmitoylation for either mutant compared to wild-type GluN2B (Figure 6A,B). In future experiments, it would be interesting to more precisely compare stoichiometry of palmitoylation of GluN2B cysteine residues in FVB/N vs. YAC128 striatal tissue.

Although we found that HIP14 interacts with GluN2B lacking either one of the two Cys clusters, it appears to palmitoylate only Cys cluster I, as evidenced by the enhanced palmitoylation and perinuclear accumulation of Cys cluster I (GluN2B 5CS)- but not Cys cluster II (GluN2B 3CS)-containing NMDARs, when transfected in COS cells (Table 1). Surprisingly, however, *Hip14*-/- mice striatal tissue did not show reduced palmitoylation of GluN2B. Instead, enhanced GluN2B palmitoylation was observed, suggesting either that: other PATs can palmitoylate GluN2B Cys cluster I in the striatum, such as zDHHC3 (Hayashi et al., 2009); the 10% residual HIP14 activity (Young et al., 2012) is sufficient to maintain cluster I palmitoylation; and/or that palmitoylation on cluster II is upregulated when cluster I palmitoylation is reduced, as suggested in HIP14L-transfected COS cells where absence of Cys cluster I resulted in increased palmitoylation on cluster II. Moreover, absence of palmitoylation of Cys cluster I (GluN2B 3CS) did not alter the surface expression of 2B-NMDARs in striatal neurons in MSN-CTX co-cultures from either FVB/N or YAC128 mice. Consistent with the increase rather than decrease in total GluN2B palmitoylation found in striatum of mice with constitutive HIP14 deficiency, and lack of effect of palmitoylation-resistant Cys cluster I GluN2B on 2B-NMDAR surface expression, acute knockdown of endogenous HIP14 by *Hip14* ASO did not affect the surface expression of GluN2B or NMDAR whole-cell currents (data not shown) recorded from striatal neurons in MSN-CTX co-cultures from either FVB/N or YAC128 mice. Taken together, these results suggest that HIP14-regulated palmitoylation of GluN2B Cys cluster I does not alter synaptic trafficking and function of 2B-NMDARs in striatal neurons in either FVB/N wild-type or YAC128 HD mice. Indeed, postsynaptic NMDAR numbers and NMDA:AMPA receptor ratios were not affected in both striatal and hippocampal neurons of *Hip14*-/- mice (Milnerwood et al., 2013). In addition, the loss of striatal neurons in *Hip14*-/- mice occurs during embryonic development (Singaraja et al., 2011), so is unlikely related to 2B-NMDAR subcellular distribution, since extrasynaptic 2B-NMDARs do not mediate toxic signaling in early development (Hardingham and Bading, 2002). On the other hand, progressive impairment of HIP14 function with age and later disease stage in YAC128 mice (Singaraja et al., 2011; Milnerwood et al., 2013) could impact GluN2B palmitoylation on Cys cluster I in cortex, with potential consequences for 2B-NMDAR synaptic distribution and function in cortical neurons (Hayashi et al., 2009; Mattison et al., 2012), which was not tested here.

Intriguingly, HIP14L did not palmitoylate either wild-type GluN2B or GluN2B lacking Cys cluster II (GluN2B 5CS) but did palmitoylate and alter subcellular distribution of GluN2B lacking cluster I (GluN2B 3CS) in COS cells (Table 1). These results suggest that in a heterologous overexpression system, palmitoylation on Cys cluster I – whether by HIP14 or another PAT such as zDHHC3, for which GluN2B is a known substrate (Hayashi et al., 2009) – alters the conformation of the GluN2B C-terminus to reduce access by HIP14L and thereby inhibit its palmitoylation of Cys cluster II (though another PAT, such as zDHHC3 that is endogenously expressed in COS cells, may still be capable of palmitoylating Cys cluster II). As discussed above, it has been reported that reduced Cys cluster I palmitoylation impairs synaptic localization of 2B-NMDARs in cortical neurons (Hayashi et al., 2009). Thus, it is possible that, in those neurons, under conditions of reduced cluster I palmitoylation, the facilitation of HIP14L-mediated cluster II palmitoylation acts as a brake to decrease delivery of 2B-NMDAR to the surface and prevent excess extrasynaptic receptor expression.

The fact that HIP14L can act as a PAT for GluN2B Cys cluster II suggests it is involved in the altered GluN2B palmitoylation and trafficking we found in YAC128 striatal neurons (Figure 8). Although HIP14L activity and palmitoylation level was not determined in striatum of YAC128 mice due to the lack of available HIP14L-specific antibodies, reduced HIP14L interaction with mutant versus wild-type Htt protein has been shown in transfected non-neuronal cells (Sutton et al., 2013). Notably, HIP14 and HIP14L interact with the same binding domain in the same orientation in Htt protein (Sanders et al., 2014), and the interaction is essential for HIP14 PAT activity (Huang et al., 2011; Sanders et al., 2014); moreover, loss of wild type Htt, or presence of mHtt in YAC128 mice, leads to the dysfunction of HIP14 (Huang et al., 2011; Singaraja et al., 2011). Those results, combined with our data showing significant reduction of GluN2B palmitoylation in striatum of *Hip14L*-/- mice, suggest the possibility that HIP14L activity is reduced in striatum of YAC128 mice (Figure 8), contributing to the reduction in GluN2B Cys cluster II palmitoylation. The importance of HIP14L as a PAT for Cys cluster II of GluN2B was further confirmed with knockdown of endogenous HIP14L in culture, which enhanced the surface/internal ratio of GluN2B in wild-type striatal MSNs, an effect that was occluded in YAC128 MSNs. Furthermore, knockdown of endogenous HIP14L led to increased susceptibility of striatal neurons to NMDA neurotoxicity (Figure 7F,G), providing a direct link between enhanced extrasynaptic localization of 2B-NMDAR, as a result of reduction of GluN2B cluster II palmitoylation, and neuronal vulnerability to NMDA toxicity.

In addition to reduced HIP14L function, what are some other mechanisms by which mHtt could reduce palmitoylation of 2B-NMDAR, and consequently enhance its surface and extrasynaptic distribution and function? If there is cross-talk between phosphorylation at specific sites in the GluN2B C-terminal domain and palmitoylation on Cys cluster II, then altered GluN2B palmitoylation and trafficking could also result from mHtt effects on protein kinases or phosphatases. Precedence for such crosstalk has been shown previously for AMPA-type glutamate receptors (Hayashi et al., 2005), for Cys cluster I of GluN2B (Hayashi et al., 2009), and for spliced stress-regulated exon (STREX) variant of large conductance calcium- and voltage- activated potassium (BK) channels (Tian et al., 2010). Notably, mHtt has been shown to alter the expression and/or activity of protein kinases and phosphatases that are known to regulate phosphorylation in the GluN2B C-terminal domain (Fan et al., 2008; Xifró et al., 2009; Metzler et al., 2010; Sanz-Clemente et al., 2010, 2013). Alternatively, if there were chronic upregulation of excitatory synaptic activity onto striatal neurons associated with mHtt expression in YAC128 mice, this could drive a decrease in GluN2B palmitoylation, as we found after 4 h of 4-AP/picrotoxin treatment (R. Kang and L. A. Raymond, Unpublished data). Consistent with this idea, one study showed enhanced cortical glutamate release onto striatal neurons in brain slice from 1 to 2 month-old YAC128 mice (Joshi et al., 2009). Further work is required to determine the precise mechanism(s) of reduced GluN2B palmitoylation in relation to enhanced extrasynaptic distribution that is associated with mHtt expression.

In this study, we found that zDHHC17 (HIP14) and zDHHC13 (HIP14L) differentially regulate Cys cluster I and II palmitoylation of GluN2B (Figure 8), respectively, adding to mechanistic knowledge of NMDAR regulation by palmitoylation. Notably, elegant studies by Lemonidis et al. (2015) showed that the ankyrin repeat (AR) domains of HIP14 and HIP14L recognize a novel unstructured peptide sequence, having a [VIA][VI]XXQP motif, which is found in the C-terminal region of GluN2B. Although unusual, our finding of selective palmitoylation by different PATs on two Cys clusters in GluN2B is not the first demonstration of targeted palmitoylation by different PATs at specific cysteines in the same substrate protein (Tian et al., 2010).

GluN2B palmitoylation is reduced in the striatum but not cortex of 1 month-old YAC128 mice, correlating with the greater susceptibility of striatal neurons to early degeneration in HD. The fact that turnover of GluN2B palmitoylation is relatively rapid, on the order of a few hours (Figure 1, Hayashi et al., 2009), suggests this mechanism could be targeted for treatment in early HD. One approach could be agents that inhibit acyl palmitoyl thioesterases (APT), enzymes that remove palmitate from proteins (Lin and Conibear, 2015; Yokoi et al., 2016). Several APT inhibitors have been developed (Adibekian et al., 2011), and the numbers of newly identified neuronal acyl palmitoyl thioesterases with selective substrate specificity are expanding along with our knowledge of the serine hydrolase superfamily to which APTs belong (Bachovchin et al., 2010; Yokoi et al., 2016). Further work is required to identify the APT(s) for which GluN2B is a substrate, and test selective APT inhibition as a potential therapeutic in HD mouse models.

## Author Contributions

RK and LR conceived and coordinated the study and wrote the manuscript. RK performed and analyzed the experiments. LW performed the experiments shown in Figure 1D,E, 7C,D. SS performed and analyzed the experiments shown in Supplementary Figure S1 and dissected *Hip14*-/-, *Hip14L*-/- mice brains for biochemical analysis. KZ performed and analyzed the experiments shown in Figure 5C,D. All authors reviewed the results and approved the final version of the manuscript.

## Conflict of Interest Statement

The authors declare that the research was conducted in the absence of any commercial or financial relationships that could be construed as a potential conflict of interest.

## Footnotes

1 http://rsb.info.nih.gov/ij/plugins/colocalization.html

## Abbreviations

2-BP: 2-Bromo palmitate
3′UTR: three prime untranslated region
ANOVA: analysis of variance
ARD: Ankyrin repeat domain
ASO: antisense oligonucleotide
Btn-BMCC: 1-biotinamido-4-[4′- (maleimidomethyl) cyclohexanecarboxamido] butane, biotin–HPDP (Biotin-HPDP-*N*-[6-(Biotinamido)hexyl]-3′-(2′-pyridyldithio) propionamide
DHHC: aspartate-histidine-histidine-cysteine
DIV: days *in vitro;*
Flag: EDTA: ethylenediaminetetraacetic acid
FVB/N: Friend Virus B/N strain
GFP: green fluorescent protein
HAM: hydroxylamine
HD: Huntington Disease
HIP14: huntingtin interacting protein
HIP14L: huntingtin interacting protein-like
Htt: huntingtin protein
mHtt: mutant huntingtin protein
NEM: *N*-ethylmaleimide
NMDAR: *N*-methyl-D-aspartate receptors
PAT: palmitoyl acyltransferase
SDS-PAGE: sodium dodecyl sulfate polyacrylamide gel electrophoresis
SNARE: neuronal *N*-ethylmaleimide-sensitive-factor-attachment-protein receptor
TUNEL: terminal deoxynucleotidyl transferase-mediated dUTP nick end labeling
YAC128: yeast artificial chromosome containing 128 CAG repeats
