# Supplementary material for: Altered Regulation of Striatal Neuronal N-Methyl-D-Aspartate Receptor Trafficking by Palmitoylation in Huntington Disease Mouse Model
Source: Front Synaptic Neurosci. 2019 Feb 21;11:3. doi: 10.3389/fnsyn.2019.00003 (PMC6393405; doi:10.3389/fnsyn.2019.00003)
Supplement: Supplementary file 1 [file Data_Sheet_1.docx]

**Supplementary figures**

For

**Altered regulation of striatal neuronal N-methyl-D-aspartate receptor trafficking by palmitoylation in Huntington disease mouse model**

Rujun Kang^1^, Liang Wang^1^, Shaun S. Sanders^2^, Kurt Zuo^1^, Michael R. Hayden^2^, Lynn A. Raymond^1^

Includes:

Supplementary Figure 1

Supplementary Figure 2

Supplementary Figure 3

**Supplementary Figure 1- Dose and time dependent knockdown of endogenous HIP14 with *Hip14* antisense oligonucleotide (ASO)**

FVB/N mouse cortical neurons were treated on DIV3 with various doses of a *Hip14* ASO (in nM): 0, 31.3, 62.5, 125, 250, or 500. Neurons were harvested 2, 4, 7 and 11days post-treatment and HIP14 protein levels were assessed by western blot. Representative blot shows knock-down of endogenous HIP14 protein expression in a dose-dependent manner. Quantification graph of HIP14 levels relative to the loading control, β-tubulin and normalized to untreated condition, from three independent experiments is shown.

**Supplementary Figure 2 – Verification of HIP14L siRNA in COS-7 cells**

COS-7 cells were co-transfected with Flag-tagged HIP14L or HIP14 with or without pSuper-HIP14L siRNA or pSuper-GFP HIP14L siRNA in 6 well plates; pSuper-scrambled siRNA and pSuper-GFP-scrambled siRNA were used as controls. The co-transfection ratio was 1:1. After 36-48 hours of overexpression, cells were harvested and HIP14L and HIP14 protein expression was assessed by western blot. Representative blots show specific knock-down of overexpressed HIP14L protein expression (A) without altering HIP14 protein expression (B). Each of the representative western blot panels shown in the figures is an example from one blot; the dividing line in each panel indicates where some lanes on the original blot were removed to display specific results in a side-by-side comparison.


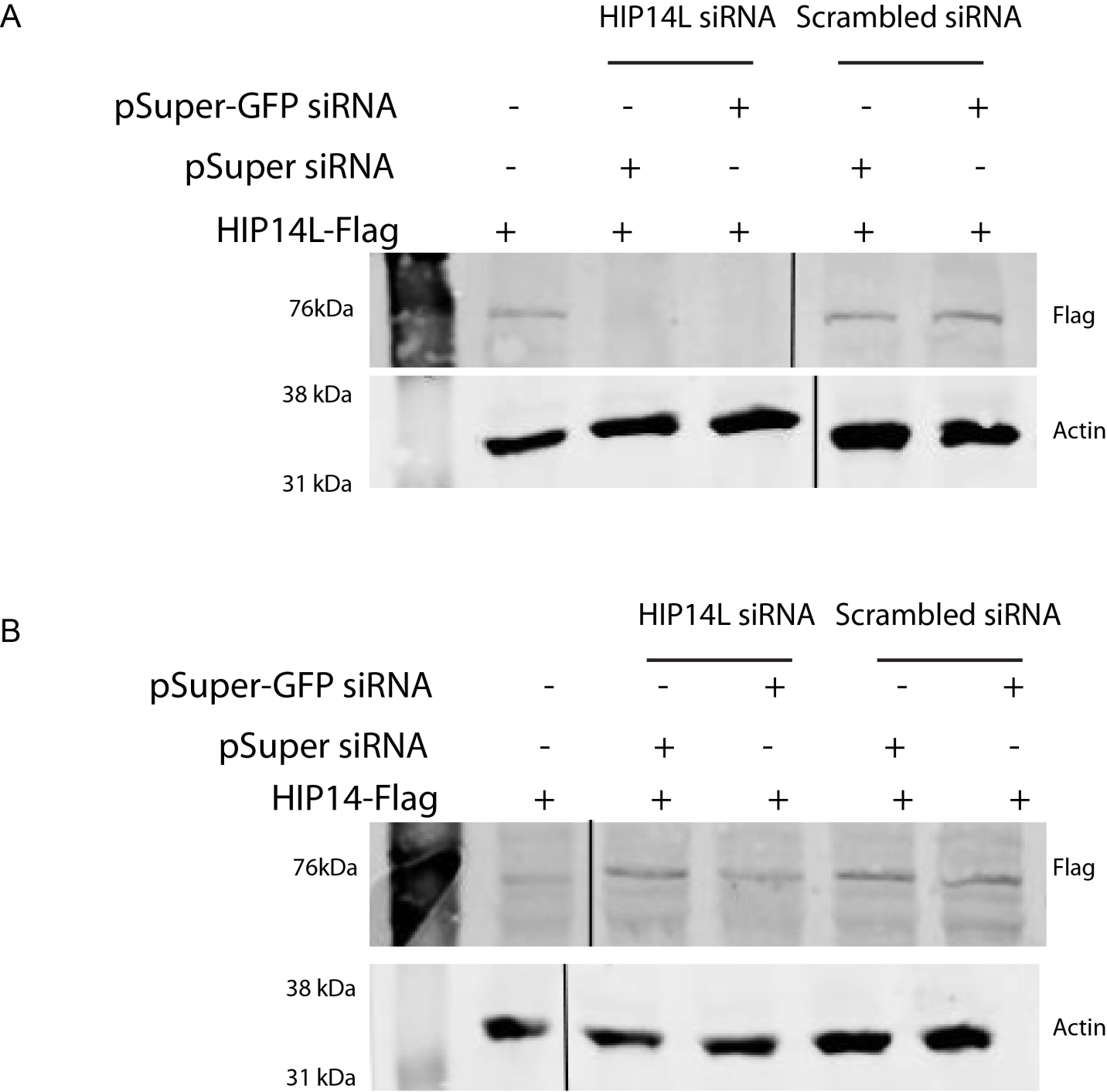


Supplementary Figure 3 – Representative images of surface vs internal expression of GluN2B with or without *Hip14* ASO or *Hip14L* siRNA treatment

(A, B): Striatal neurons in MSN-CTX co-cultures from FVB/N (A) and YAC128 (B) mice were nucleofected with GFP-tagged GluN2B WT then treated with 250 nM *Hip14* ASO for 10 days from DIV 4 to DIV 14. Cultures were then live-stained for surface GluN2B (Green) with GFP antibody, and then fixed and stained for internal GluN2B (Red). A merged image shows the total GluN2B expression. (C, D):GFP-tagged GluN2B WT with or without *Hip14L* siRNA were nucleofected in the MSN co-cultured with CTX from FVB/N (C) and YAC128 (D) mice. Images represent surface (green) /internal (Red) expression of GluN2B in striatal MSN from DIV 14 MSN-CTX cultures.
